# Identification of patient subtypes based on protein expression for prediction of heart failure after myocardial infarction

**DOI:** 10.1016/j.isci.2023.106171

**Published:** 2023-02-11

**Authors:** Wilfried Heyse, Vincent Vandewalle, Guillemette Marot, Philippe Amouyel, Christophe Bauters, Florence Pinet

**Affiliations:** 1 UniversityLille, Inserm, CHU Lille, Institut Pasteur de Lille, U1167 - RID-AGE - Facteurs de risque et déterminants moléculaires des maladies liées au vieillissement, 59000 Lille, France; 2 UniversityLille, CHU Lille, URL2694-METRICS - Evaluation des technologies de santé et des pratiques médicales, 59000 Lille, France; 3 Inria, Modal, 59000 Lille, France

**Keywords:** Cardiovascular medicine, Proteomics

## Abstract

This study investigates the ability of high-throughput aptamer-based platform to identify circulating biomarkers able to predict occurrence of heart failure (HF), in blood samples collected during hospitalization of patients suffering from a first myocardial infarction (MI). REVE-1 (derivation) and REVE-2 (validation) cohorts included respectively 254 and 238 patients, followed up respectively 9 · 2 ± 4 · 8 and 7 · 6 ± 3 · 0 years. A blood sample collected during hospitalization was used for quantifying 4,668 proteins. Fifty proteins were significantly associated with long-term occurrence of HF with all-cause death as the competing event. *k*-means, an unsupervised clustering method, identified two groups of patients based on expression levels of the 50 proteins. Group 2 was significantly associated with a higher risk of HF in both cohorts. These results showed that a subset of 50 selected proteins quantified during hospitalization of MI patients is able to stratify and predict the long-term occurrence of HF.

## Highlights

• We quantified 4,668 proteins in two cohorts of patients with a first myocardial infarction
• Fifty proteins were significantly associated with the occurrence of heart failure
• A clustering method identified two groups of patients based on the 50 proteins
• Group 2 of patients was associated with a high risk of occurrence of heart failure

## Graphical abstract

**Figure:**
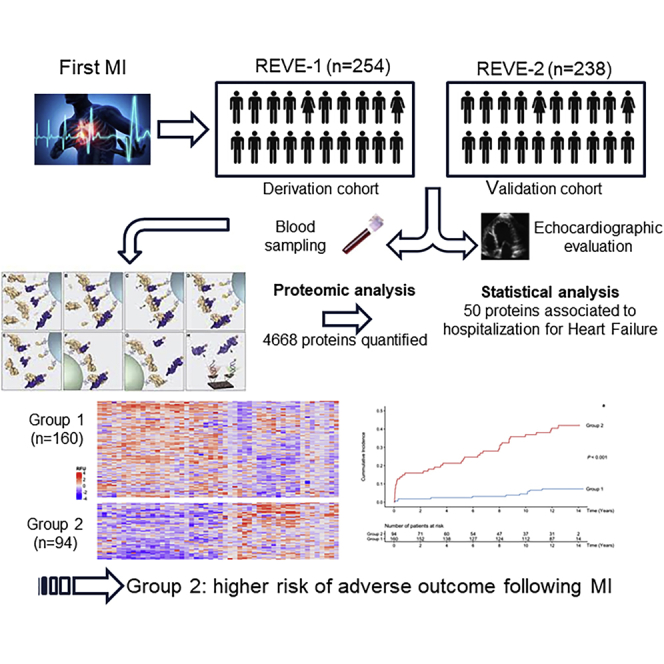

## Introduction

Despite significant therapeutic improvements during the last decades, the long-term risk of heart failure (HF) after myocardial infarction (MI) remains significant and ischemic HF is a major cause of mortality worldwide.^1^ Post-MI HF is too often diagnosed at a late stage when its irreversible consequences are already established. The estimation of the risk of HF in the early post-MI period currently relies on clinical variables, left ventricular function parameters, and conventional cardiac biomarkers such as troponin or B-type natriuretic peptide (BNP). However other cardiovascular biomarkers might reflect the activation of new potential pathways after MI and contribute to HF in both the short and long term.^2^ Broader approaches are important for clinicians to understand the implications of different pathobiological axes. Despite the proliferation of candidate biomarkers, there is limited data comparing comprehensively the prognostic value of biomarkers when assessed in large arrays.

Explorative analysis using large scale protein measurement methods allow the simultaneous analysis of large biomarker panels. Thanks to SOMA(Slow Off-rate Modified Aptamers)scan assay, over 5,000 proteins can be measured covering 8 logs of abundance in the human proteome. These proteins are not targeted toward any particular disease and, thus, such wide panels of proteins can help to discover new biomarkers using appropriate statistical methods to analyze these data. Very recently, aptamer assays were shown to provide excellent precision and an unprecedented coverage and promise for disease associations.^3^ Using SOMAscan profiling (4,453 targets), Gui et al. showed that plasma multiprotein score improved risk stratification in patients with HF and reduced ejection fraction and identified novel candidates.^4^

**Figure 1:**
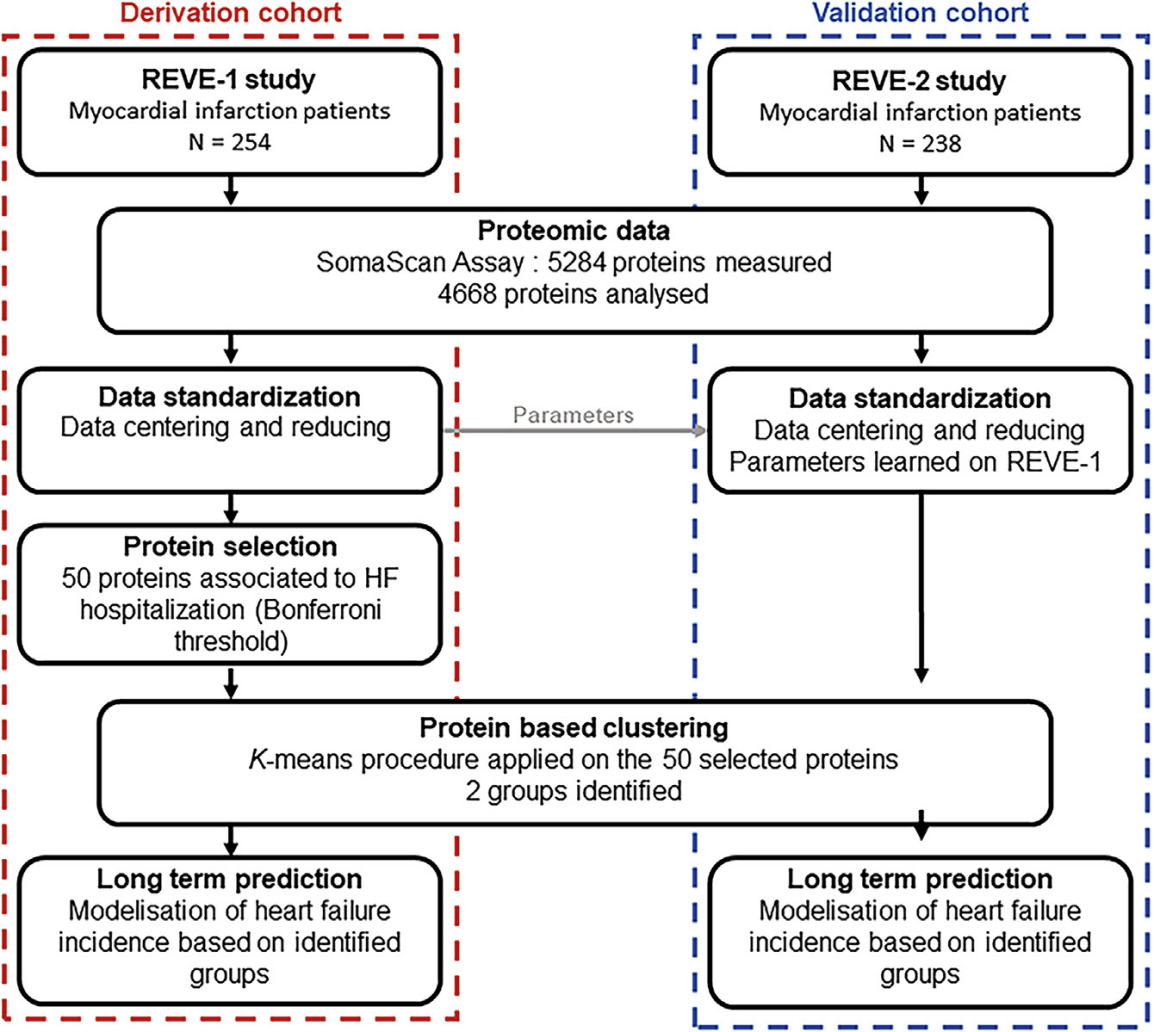
Overview of the study REVE-1 study^5^ was used as derivation cohort and REVE-2 study^6^ as validation cohort. Proteomic data analysis was performed on all plasma samples collected during hospitalization of patients from both cohorts by SOMAscan assay (version V4.0). The SOMAScan platform measured accurately 4668 plasma proteins. Log2 transformed data were centered and reduced. Standardization parameters were calculated with the data from the REVE-1 cohort (see Table S2) and then applied to both REVE-1 and REVE-2 cohorts. Univariate competing risk models were fitted for each protein, and significance tests selected 50 proteins to be associated with occurrence of hospitalization for HF (p< 1 · 07 10^−5^ in accordance with Bonferroni’s method). Clustering was then performed on the REVE-1 patients with the 50 selected proteins, by *k-*means procedure with k = 2 groups. Patients of REVE-2 were then assigned to one of the groups identified on REVE-1. A competing risk model was then developed based on the group information on both cohorts.

The aim of this work was to use circulating plasma proteins expression levels quantified during the hospitalization of patients suffering from MI to identify groups of patients able to predict the long-term risk of occurrence of HF after MI. The prospective REVE-1 (REmodelage VEntriculaire) cohort^5^ and REVE-2 cohort^6^ in which patients with a first MI were included and underwent long-term follow-up^7^ were used respectively as derivation and validation cohorts. A large panel of 4,668 proteins was measured with the SOMAscan assay in blood sample collected during initial hospitalization. To build groups of patients able to predict the occurrence of HF, the analysis was divided into three steps (Figure 1). First, a protein selection step was performed to focus on the relevant proteins in the derivation cohort, REVE-1. Second, the selected proteins were used to build groups of patients on REVE-1 using *k*-means, an unsupervised clustering algorithm. Patients of REVE-2, the validation cohort, were assigned to the groups built on REVE-1. Third, the groups’ predictive ability for occurrence of hospitalization for HF was assessed using competing risk models in both cohorts. In addition, Ingenuity Pathway Analysis (IPA) and Gene Ontology (GO) analysis were performed to define molecular networks enriched from the selected proteins.

### Evidence before this study

HF following an MI is too often diagnosed too late when its irreversible consequences are established. The estimation of the risk of HF in the early post-MI period currently relies on clinical variables, left ventricular function parameters, and conventional cardiac biomarkers such as troponin or BNP. Despite proliferation of candidate biomarkers, there is limited data comparing comprehensively the prognostic value of biomarkers.

### Added value of this study

We performed a discovery proteomics approach by quantification of 4,468 proteins in two cohorts of patients with a first MI, REVE1 (derivation cohort) and REVE 2 (validation cohort). A total of 50 proteins were selected to be significantly associated with the occurrence of hospitalization for HF. An unsupervised clustering method identified 2 groups of patients based on the expression levels of the 50 proteins in the REVE1 cohort that were validated in the REVE2 cohort. Differences in protein expression led to identifying key physiopathological processes combining differences in molecules leading to two groups of patients, low (group 1) and high (group 2) risk of long-term adverse cardiac outcomes following MI.

### Implications of the available evidence

Based on the expression of the 50 proteins, group 2 of patients was associated with a high risk of occurrence of HF. Stratification of MI patients based on proteins involved in cell-to-cell communication should be important in the future.

## Results

### Study populations

Patients in both cohorts had similar age and gender; indicators of MI size (wall motion score index and peak creatine kinase) and Killip class were also similar (Table 1). Patients in the REVE-2 cohort were more often treated by primary PCI and less often by thrombolysis. Statistical differences were observed between both cohorts for diastolic blood pressure, heart rate, end systolic volume, end diastolic volume and wall motion systolic index. The mean follow-up was 9 · 2 ± 4.8 years in REVE-1 and 7.6 ± 3.0 years in REVE-2. One patient was lost during the follow-up leading to 254 patients in REVE-1 for the analysis; no patients in REVE-2 were lost during the follow-up. The numbers of patients who reached the primary endpoint (hospitalization for HF) during follow-up were respectively 49 in REVE-1 and 28 in REVE-2. The numbers of patients who reached the competing event (death from all causes) during the long term follow-up were respectively 63 in REVE-1 and 26 in REVE-2.

**Table 1:** Baseline characteristics of the patients included in the Derivation (REVE-1) and Validation (REVE-2) cohorts

	REVE-1 (n = 254)	REVE-2 (n = 238)	p value
Age (years)	58 ± 14	57 ± 14	0.28
Women, n (%)	66 (26)	46 (19)	0.23
Body mass index (kg/m^2^)	27 · 1 ± 4 · 8	27 · 2 ± 4 · 7	0.83
Hypercholesterolemia, n (%)	119 (47)	79 (33)	0.48
History of hypertension, n (%)	114 (45)	86 (36)	0.46
Smoking, n (%)	173 (68)	164 (69)	0.68
Diabetes mellitus, n (%)	58 (23)	51 (21)	0.19
Initial reperfusion therapy, n (%)			
Primary PCI	76 (30)	124 (52)	**<0.001**
Thrombolysis	133 (52)	85 (36)	**<0.001**
None	45 (18)	29 (12)	0.11
Multivessel CAD, n (%)	96 (39)	94 (41)	0.39
PCI during hospitalization, n (%)	226 (89)	205 (86)	0.91
Final TIMI grade 3 flow in infarct-related vessel, n (%)	211 (84)	206 (90)	0.84
Systolic blood pressure (mmHg)	112 ± 16	110 ± 15	0.24
Diastolic blood pressure (mmHg)	66 ± 12	63 ± 11	**<0.001**
Heart rate (bp.m.)	68 ± 12	72 ± 14	**0.01**
Killip class ≥2, n (%)	70 (28)	76 (32)	0.25
Peak creatine kinase (IU/L)	2558 [1252 to 4013]	2348 [1450 to 4192]	0.48
End-systolic volume (mL/m^2^)	29 ± 11	27 ± 10	**0.01**
End Diastolic Volume (mL/m^2^)	57 ± 15	52 ± 14	**<0.001**
Left ventricular ejection fraction (%)	49 ± 10	49 ± 8	0.58
Wall motion score index	1.87 ± 0.15	1.91 ± 0.15	**<0.001**
Treatment at discharge, n (%)			
Antiplatelet therapy	247 (98)	235 (99)	1
β-blockers	238 (94)	230 (97)	1
ACE-I or ARB	244 (96)	230 (97)	1
Aldosterone antagonists	34 (13)	78 (33)	0.05
Statins	248 (98)	225 (95)	1

Data are presented as mean ± SD frequency (percentage) or median [Q1 to Q3].

bpm, beats per minute; CAD, coronary artery disease; PCI, percutaneous coronary intervention; TIMI, thrombolysis in myocardial infarction; ACE-I, angiotensin-converting enzyme inhibitors; ARB, angiotensin II receptor blockers. Imputed data are not shown in this table.

### Selection of proteins

Standardization parameters for each protein and each patient were calculated using the mean and standard deviation (SD) calculated with the data from REVE-1 cohort (Table S2). The proteomic data of REVE-2 were then standardized protein by protein for each patient using these values following the calculation:((value of the protein) – (the mean of protein in REVE-1))/(the corresponding SD in REVE-1).

After standardization, 50 proteins were selected to be significantly associated with the outcome event in REVE-1 (listed in Table 2 with their significance levels and subhazard ratios and in Table S3 for their location and type). Among the 50 proteins selected on REVE-1, 44 proteins on REVE-2 were regulated in the same manner for the outcome of REVE-2 patients and 6 were conversely but not significantly regulated (PDE4A, SAMHD1, RFNG, ST3GAL5, NMS and DPP4) (Table 2). The correlation heatmap among the 50 selected proteins in REVE-1 shows that some of the proteins are highly correlated, with the highest correlation of 0.893 between B2M and CST3 (Figure S2). This shows that several proteins carry a close tendency, which will be taken into account by the following clustering approach.

**Table 2:** List of plasma proteins selected to be associated with occurrence of heart failure in REVE-1 cohort

REVE-1		REVE-2		UniProt ID	Symbol	Entrez gene name
p value^a^	SHR#	p value^a^	SHR#			
<1·0e-16	1 · 18	3·1e-03	2 · 59	C9JXX5	C11ORF94	Uncharacterized protein C11orf94
3·8e-12	1 · 17	5·0e-01	0 · 71	P27815	PDE4A	cAMP-specific 3′,5′-cyclic phosphodiesterase 4A
9·0e-11	1 · 31	9·8e-01	0 · 99	Q9Y3Z3	SAMHD1	Deoxynucleoside triphosphate triphosphohydrolase SAMHD1
2·0e-10	1 · 16	8·6e-01	0 · 97	Q9Y644	RFNG	Beta-1,3-N-acetylglucosaminyltransferase radical fringe
2·4e-10	0 · 41	1·1e-03	0 · 50	O75473	LGR5	Leucine-rich repeat-containing G-protein coupled receptor 5
1·5e-08	1 · 12	4·9e-01	1 · 19	Q16568	CARTPT	Cocaine- and amphetamine-regulated transcript protein
1·8e-08	0 · 52	4·8e-04	0 · 57	P56704	WNT3A	Protein Wnt-3a
2·6e-08	0 · 60	1·5e-02	0 · 65	P05543	SERPINA7	Thyroxine-binding globulin
3·6e-08	0 · 57	5·8e-02	0 · 85	P19823	ITIH2	Inter-alpha-trypsin inhibitor heavy chain H2
4·0e-08	1 · 54	3·3e-02	1 · 21	Q8TAT2	FGFBP3	Fibroblast growth factor-binding protein 3
4·8e-08	1 · 15	3·2e-04	2 · 18	Q92831	KAT2B	Histone acetyltransferase KAT2B
9·8e-08	1 · 83	1·6e-04	2 · 06	Q9Y251	HPSE	Heparanase
1·5e-07	1 · 32	2·0e-03	1 · 25	A8K7I4	CLCA1	Calcium-activated chloride channel regulator 1
2·7e-07	0 · 52	1·4e-03	0 · 47	Q9NTU7	CBLN4	Cerebellin-4
3·6e-07	1 · 78	2·4e-04	1 · 58	P61769	B2M	Beta-2-microglobulin
3·7e-07	1 · 48	9·1e-05	1 · 73	Q2I0M5	RSPO4	R-spondin-4
5·2e-07	1 · 33	4·3e-02	1 · 36	P52943	CRIP2	Cysteine-rich protein 2
9·5e-07	0 · 59	4·7e-01	0 · 87	P33764	S100A3	Protein S100-A3
*1.3e-06*	*1.50*	*3*·*6e-01*	*1*·*22*	*Q9BUP3*	*HTATIP2*	*Oxidoreductase HTATIP2*
1·4e-06	0 · 40	7·9e-05	0 · 41	Q96PQ0	SORCS2	VPS10 domain-containing receptor SorCS2
1·4e-06	0 · 55	3·5e-02	0 · 68	Q8IWT1	SCN4B	Sodium channel subunit beta-4
1·5e-06	0 · 48	8·8e-04	0 · 59	Q6P988	NOTUM	Palmitoleoyl-protein carboxylesterase NOTUM
1·7e-06	0 · 57	2·9e-05	0 · 54	P29622	SERPINA4	Kallistatin
1·9e-06	1 · 79	6·7e-05	1 · 75	P01034	CST3	Cystatin-C
1·9e-06	2 · 71	5·2e-01	1 · 21	Q8N6C8	LILRA3	Leukocyte immunoglobulin-like receptor subfamily A member 3
2·0e-06	0 · 23	6·4e-01	1 · 05	Q9UNP4	ST3GAL5	Lactosylceramide alpha-2,3-sialyltransferase
2·7e-06	0 · 50	1·5e-07	0 · 42	Q8N6M8	IQCF1	IQ domain-containing protein F1
3·1e-06	1 · 28	3·2e-02	1 · 60	P0DJ93	SMIM13	Small integral membrane protein 13
3·2e-06	0 · 68	7·1e-02	0 · 89	P01008	SERPINC1	Antithrombin-III
3·4e-06	1 · 87	4·1e-06	2 · 86	Q8TE54	SLC26A7	Anion exchange transporter
3·4e-06	0 · 41	8·0e-01	0 · 87	Q6ZUB0	SPATA31D4	Putative spermatogenesis-associated protein 31D4
4·3e-06	1 · 83	5·6e-04	1 · 72	P16860	NPPB	Natriuretic peptides B
4·4e-06	1 · 45	5·1e-03	1 · 46	Q12805	EFEMP1	EGF-containing fibulin-like extracellular matrix protein 1
4·8e-06	0 · 55	5·7e-01	1 · 04	Q5H8A3	NMS	Neuromedin-S
5·0e-06	0 · 63	1·0e-04	0 · 65	P35858	IGFALS	Insulin-like growth factor-binding protein complex acid labile subunit
5·1e-06	1 · 39	5·4e-02	1 · 29	Q9NS68	TNFRSF19	Tumor necrosis factor receptor superfamily member 19
5·9e-06	0 · 49	1·9e-03	0 · 48	O60260	PARK2	E3 ubiquitin-protein ligase parkin
5·9e-06	1 · 34	2·5e-02	1 · 62	Q13522	PPP1R1A	Protein phosphatase 1 regulatory subunit 1A
6·4e-06	0 · 52	7·7e-02	0 · 73	P55285	CDH6	Cadherin-6
6·6e-06	0 · 58	2·7e-01	0 · 76	P45985	MAP2K4	Dual specificity mitogen-activated protein kinase kinase 4
6·7e-06	0 · 54	3·0e-07	0 · 46	Q2Y0W8	SLC4A8	Electroneutral sodium bicarbonate exchanger 1
6·8e-06	0 · 69	5·9e-02	0 · 76	P01042	KNG1	Kininogen, HMW, Two Chain
7·6e-06	0 · 49	2·5e-08	0 · 23	Q8N3Z0	PRSS35	Inactive serine protease 35
7·8e-06	0 · 51	4·0e-01	1 · 11	P27487	DPP4	Dipeptidylpeptidase 4
8·1e-06	0 · 55	5·6e-02	0 · 56	Q8IYN2	TCEAL8	Transcription elongation factor A protein-like 8
8·5e-06	1 · 75	2·5e-01	1 · 21	Q15848	ADIPOQ	Adiponectin
8·9e-06	0 · 50	4·2e-03	0 · 52	Q9UII4	HERC5	E3 ISG15--protein ligase HERC5
9·4e-06	1 · 86	1·7e-04	1 · 77	P07998	RNASE1	RNase pancreatic
9·5e-06	0 · 44	4·7e-06	0 · 39	Q9UBC7	GALP	Galanin-like peptide
9·8e-06	0 · 54	1·5e-04	0 · 49	Q9UK76	JTP1	Jupiter microtubule associated homolog 1

UniProt ID, protein symbol, full protein name are provided from the UniProtKB database (https://www.uniprot.org). The proteins in italics were quantified by several SOMAmers and the mean value was used. See also Tables S1 and S2–S6, Figures S1 and S2.

a Raw p-values are indicated, significance threshold was set at 1 · 07 10^−5^ in accordance with Bonferroni’s method; # SHR: Subhazard ratio was calculated by a competing risk model.

### Patients groups

**Figure 2:**
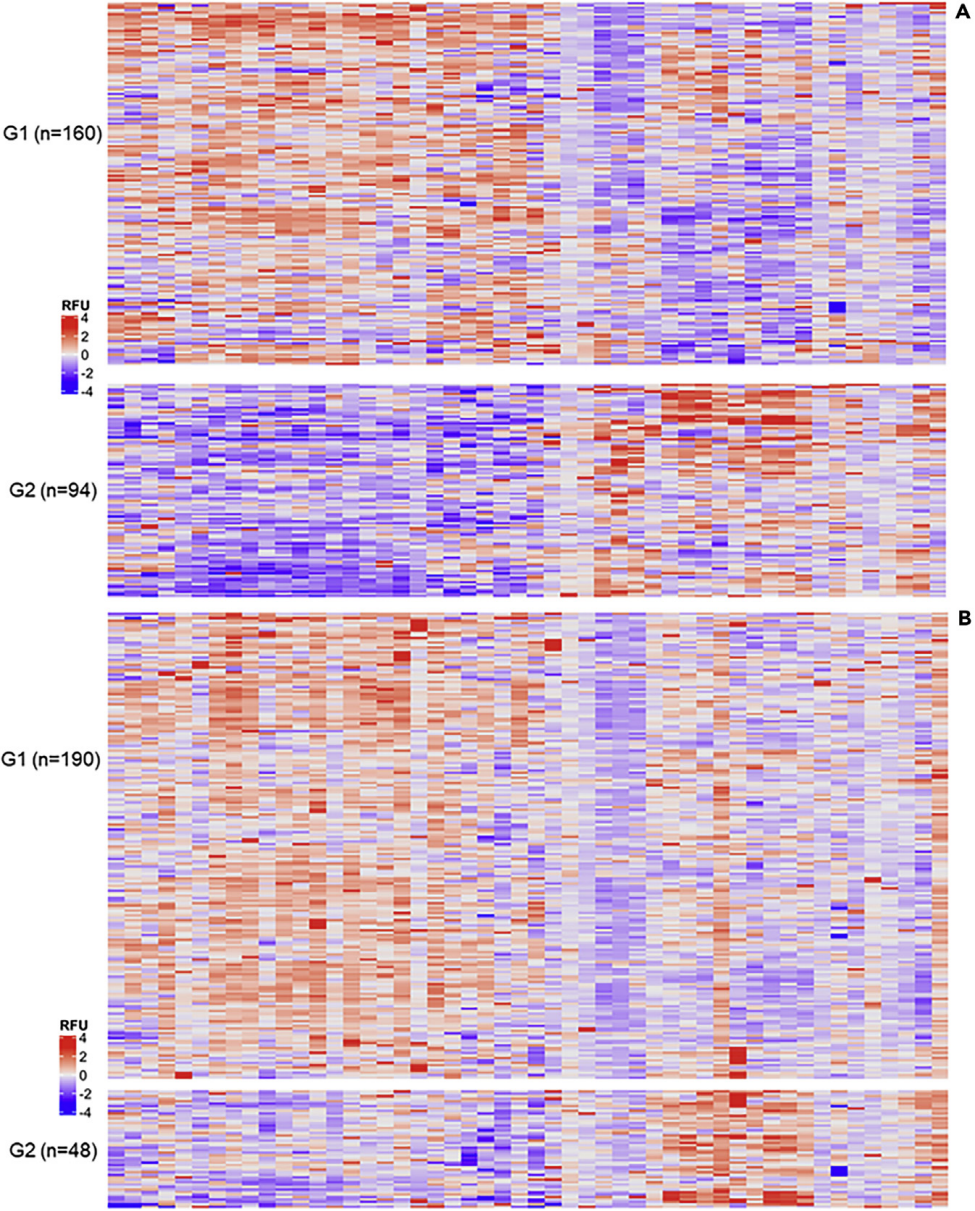
Heatmap visualization of the two identified groups on REVE-1 (A) and REVE-2 (B) cohorts Columns represent the 50 selected proteins significantly modulated according to the outcome of patients and classified in the same order for both cohorts. Lines represent the patients divided in the two groups (G1 and G2) identified by clustering with the *k*-means. Cells are colored according to protein abundance expressed in RFU (Relative Fluorescence Unit). Red represents high abundance whereas blue indicates low abundance. Protein levels are log2-transformed and standardized. See also Table S4.

The silhouette criteria led to use *k*-means procedure with k = 2 groups on REVE-1 (not shown). Patients were split as 160 patients in group 1 and 94 in group 2 and the two groups show contrasted protein expression profiles (Figure 2A).

These contrasted protein expression profiles were also found in REVE-2 cohort. Patients were mapped to one of the two groups identified on REVE-1 with respectively 190 patients assigned to group 1 and 48 to group 2 and similar characteristics were observed, with opposite protein expression profiles between the two groups (Figure 2B).

We also observed significant clinical differences between the patients of the two groups in both cohorts with significant differences in REVE-1 for history of hypertension, diabetes, initial reperfusion therapy, multi-vessel coronary artery disease (CAD), final thrombolysis in myocardial infarction (TIMI) grade 3 flow in infarct-related vessel, heart rate, Killip class over 2, end-systolic volume, left ventricular ejection fraction, WMSI and for treatments at discharge (aldosterone antagonists). In REVE-2, gender, smoking and treatments at discharge (β-blockers) were significantly different between the identified groups. Significant differences for the age were found in both cohorts. Thus, protein based clustering leads to the identification of significant differences in the clinical characteristics of patients (Table 3).

**Table 3:** Clinical characteristics of patients between the two identified groups on both cohorts

Variables	REVE-1 (Derivation) (n = 254)			REVE-2 (Validation) (n = 238)		
	Group 1 (n = 160)	Group 2 (n = 94)	p value	Group 1 (n = 190)	Group 2 (n = 48)	p value
**Age (years)**	54 ± 13	65 ± 13	**<0 · 001**	54 ± 13	68 ± 12	**<0** · **001**
***Women, n (%)***	46 (29)	20 (21)	0 · 245	30 (16)	16 (33)	***0***·***011***
Body mass index (kg/m^2^)	26.7 ± 4.7	27.6 ± 4.9	0 · 146	27 · 4 ± 4 · 4	26 · 1 ± 5 · 4	0 · 071
Hypercholesterolemia, n (%)	73 (46)	46 (49)	0 · 704	62 (33)	17 (35)	0 · 846
***History of hypertension, n (%)***	55 (34)	59 (63)	***<0***·***001***	63 (33)	23 (48)	0 · 083
***Smoking, n (%)***	110 (69)	63 (67)	0 · 884	140 (74)	24 (50)	***0***·***003***
***Diabetes mellitus, n (%)***	29 (18)	29 (31)	***0***·***029***	36 (19)	15 (31)	0 · 097
***No initial reperfusion therapy, n (%)***	22 (14)	23 (24)	***0***·***047***	19 (10)	10 (21)	0 · 071
***Multivessel CAD, n (%)***	49 (31)	47 (52)	***0***·***002***	71 (38)	23 (51)	0 · 165
PCI during hospitalization, n (%)	144 (90)	82 (87)	0 · 637	165 (87)	40 (83)	0 · 693
***Final TIMI grade 3 flow in infarct-related vessel, n (%)***	143 (90)	68 (74)	***0***·***002***	166 (90)	40 (91)	1
Systolic blood pressure (mmHg)	113 ± 16	110 ± 16	0 · 174	111 ± 14	109 ± 20	0 · 664
Diastolic blood pressure (mmHg)	67 ± 12	65 ± 12	0 · 183	63 ± 10	60 ± 15	0 · 079
***Heart rate (bpm.)***	67 ± 13	71 ± 12	***0***·***009***	72 ± 14	72 ± 14	0 · 957
***Killip class ≥ 2, n (%)***	28 (18)	42 (45)	***<0***·***001***	56 (29)	20 (42)	0 · 148
**Peak creatine kinase (IU/L)**	2259 [1160 to 3885]	2874 [464 to 4247]	0 · 100	2381 [1508 to 4211]	2078 [1291 to 3708]	0 · 554
***End-systolic volume (mL/m***^***2***^***)***	28 ± 10	32 ± 12	***<0***·***001***	27 ± 11	27 ± 10	0 · 991
End Diastolic Volume (mL/m^2^)	56 ± 15	58 ± 15	0 · 333	52 ± 14	51 ± 15	0 · 663
***Left ventricular ejection fraction (%)***	51 ± 9	45 ± 9	***<0***·***001***	50 ± 8	49 ± 8	0 · 519
***Wall motion score index***	1 · 84 ± 0 · 15	1 · 91 ± 0 · 15	***<0***·***001***	1 · 90 ± 0 · 15	1 · 93 ± 0 · 15	0 · 250
Treatment at discharge, n (%)						
Antiplatelet therapy	157 (99)	90 (96)	0 · 277	188 (99)	47 (100)	1
***ß-blockers***	152 (96)	86 (91)	0 · 288	187 (98)	43 (91)	***0***·***042***
ACE-I or ARB	153 (96)	91 (97)	1	186 (98)	44 (94)	0 · 285
***Aldosterone antagonists***	15 (9)	19 (20)	***0***·***025***	65 (34)	13 (28)	0 · 495
Statins	158 (99)	90 (96)	0 · 125	181 (95)	44 (94)	0 · 929

Results are presented as mean ± SD frequency (percentage) or median [Q1 to Q3]. The selected variables are significantly different in the two groups for both cohorts. Bold and bold italics results are significant (p< 0 · 05) respectively in both cohorts and only one cohort, with p values calculated by appropriate statistical tests (chi2 or t-test). Imputed data are not indicated in this table.

The differential expression analysis performed between the identified groups showed that 43 and 41 of the 50 selected proteins had significantly different means between the two groups respectively in REVE-1 and REVE-2 (Table S4). These results highlight that the groups identified in REVE-1 have significantly distinct proteomic expression profiles that were validated in REVE-2.

### Event prediction

**Figure 3:**
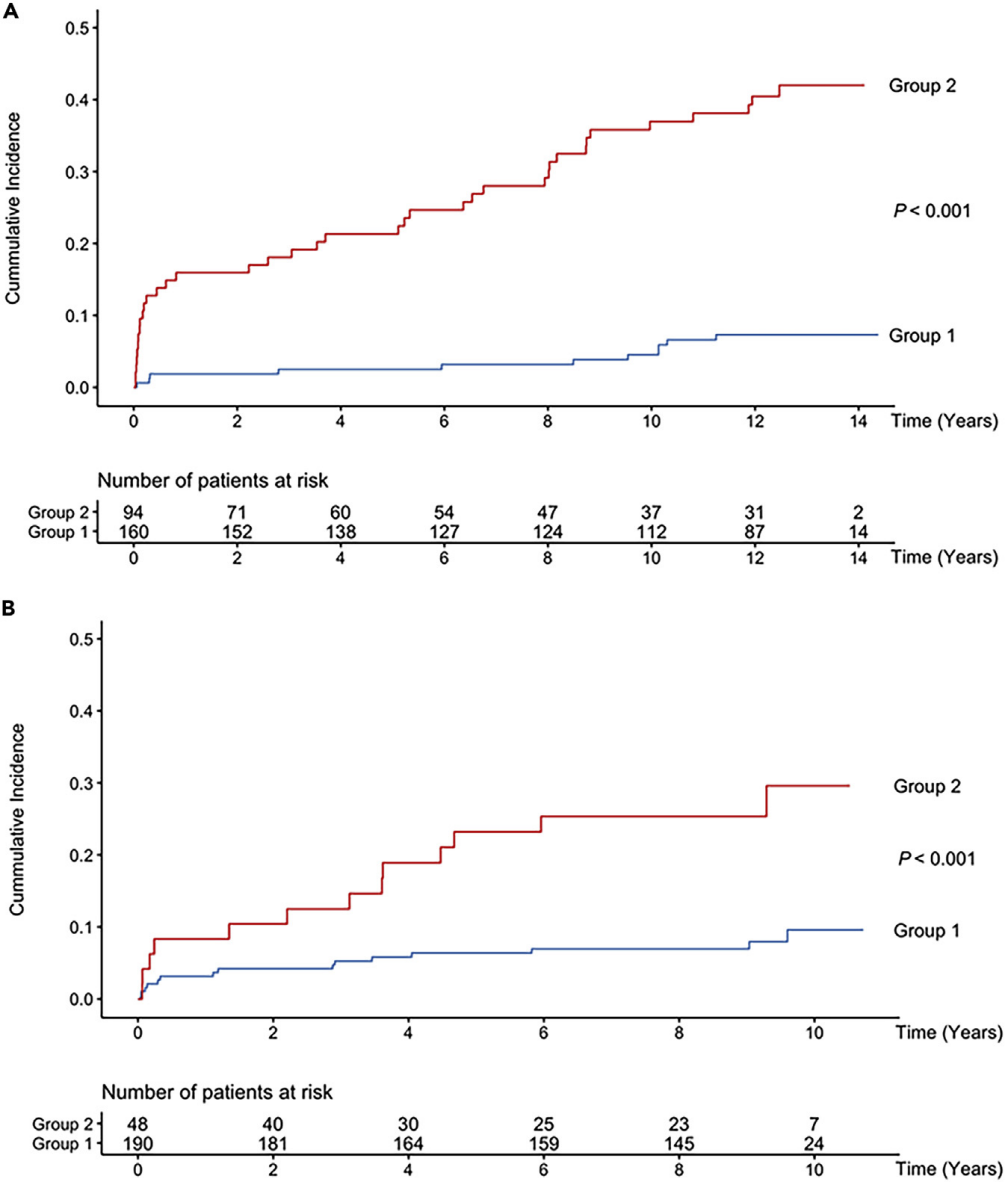
Cumulative incidence curves of HF for patients from REVE-1 (A) and REVE-2 (B) cohorts The studied populations are divided in two groups according to the two identified groups by clustering with the *k*-means. See also Table S3 and Figure S3.

Cumulative incidence curves in REVE-1 (Figure 3A) and REVE-2 (Figure 3B) showed that in both cohorts, the identified groups had distinct incidence with higher hospitalization for HF in group 2. We validated the performance of group 2 for the significantly higher risk of hospitalization for HF with an SHR of 7 · 26 ([3 · 74 - 14 · 07], p<0 · 001) in REVE-1 and 3 · 66 ([1 · 75 - 7 · 64], p<0 · 001) in REVE-2. The group’s robustness remained significant after adjustments for age, gender, ejection fraction, diabetes, Killip class, serum creatinine, BNP and NT-proBNP separately or combined in pairs. The least favorable model for the group variable was those adjusted for age and ejection fraction which showed a significant association with an SHR, for the group variable, of 4 · 50 ([2 · 19 - 9 · 26], p<0 · 001) and 2 · 19 ([1 · 05 - 4 · 93], p = 0 · 047) in REVE-1 and REVE-2, respectively (Table S5).

A sensitivity analysis was achieved by performing the clustering on the 50 selected proteins without the BNP, an established biomarker of HF. When performing the clustering on 49 proteins, only 2 patients from REVE-1 changed of groups and 1 patient form REVE-2 changed of groups. The cumulative incidence curves of these groups were very similar to those obtained with the 50 proteins (Figure S3).

### Signaling pathway analysis

**Figure 4:**
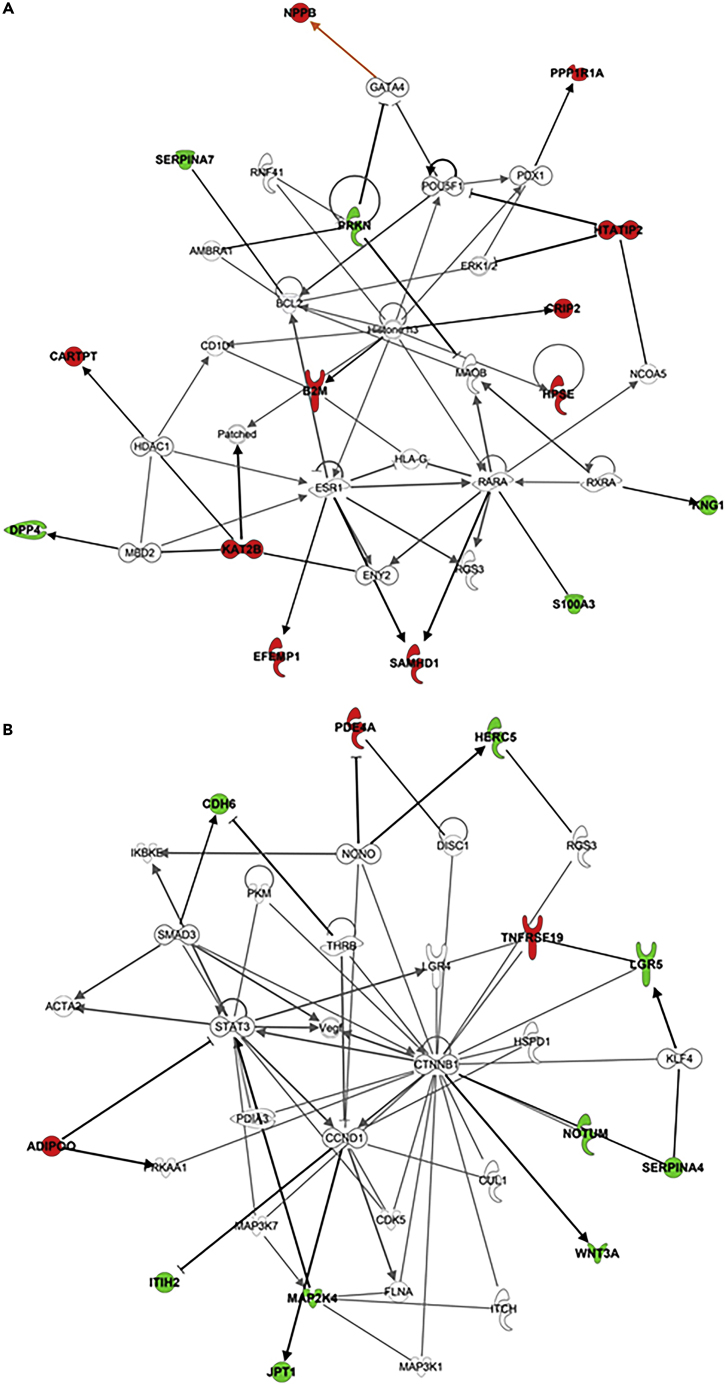
IPA based protein networks involved in worse outcome in post-MI patients of REVE-1 study The IPA analysis was performed on the 50 proteins selected to be associated with long term survival and listed in Table 2 (version: 73,620,684 Release: 2022-03-12). Only network 1 (A) and network 2 (B) from Table 4 are presented. Nodes are displayed using various shapes that represent the functional class of the proteins as published on https://qiagen.secure.force.com/KnowledgeBase/KnowledgeIPAPage?id=kA41i000000L5rTCAS. The color of proteins indicates respectively their regulated expression (red: increased and green: decreased) associated with occurrence of hospitalization for HF. The arrows indicate the modulatory effect of protein on its interacting proteins. Only direct interactions were selected. Detailed information on the molecules present in the networks is detailed in Table 4. See also Table S7.

To gain further insight into the potential mechanisms in worse outcome of post-MI patients, the 50 proteins significantly associated with occurrence of HF (Table 2) were subjected to IPA for building biological networks. Two networks ranked by a score >20, revealed a significant link with “Cell-To-Cell Signaling and Interaction, Cellular Growth and Proliferation, Gene Expression” (network 1) and “Cancer, Hair and Skin Development and Function, Organ Development” (network 2). Despite their low score, networks 8 and 9 are linked to respectively “Cardiovascular Disease, Cellular Assembly and Organization, Developmental Disorder” and “Cardiovascular Disease, Hematological Disease, Hereditary Disorder” (Table 4, Figure 4).

**Table 4:** Significant protein networks identified by IPA in REVE1

ID	Molecules in network	Score	Focus molecules	Top diseases and functions
1	AMBRA1, **B2M**, BCL2, **CARTPT**, CD1D, **CRIP2**, **DPP4**, **EFEMP1**, ENY2, ERK1/2, ESR1, GATA4, HDAC1, Histone H3, HLA-G, **HPSE**, **HTATIP2**, **KAT2B**, **KNG1**, MAOB, MBD2,NCOA5, **NPPB**, Patched, PDX1, POU5F1, **PPP1R1A**, **PRKN**, RARA, RGS3, RNF41, RXRA, **S100A3**, **SAMHD1**, **SERPINA7**	30	15	Cell-To-Cell Signaling and Interaction, Cellular Growth and Proliferation, Gene Expression
2	ACTA2, **ADIPOQ**, CCND1, **CDH6**, CDK5, CTNNB1, CUL1, DISC1, FLNA, **HERC5**, HSPD1, IKBKE, ITCH, **ITIH2**, **JPT1**, KLF4, LGR4, **LGR5**, **MAP2K4**, MAP3K1, MAP3K7, NONO, **NOTUM**, **PDE4A**, PDIA3, PKM, PRKAA1, RGS3, **SERPINA4**, SMAD3, STAT3, THRB, **TNFRSF19**, VEGF, **WNT3A**	22	12	Cancer, Hair and Skin Development and Function, Organ Development
3	FGF2, **FGFBP3**	2	1	Carbohydrate Metabolism, Drug Metabolism, Small Molecule Biochemistry
4	NFAT5, **PRSS35**	2	1	Organismal Injury and Abnormalities, Renal and Urological Disease, Renal Hydronephrosis
5	MAFB, **RNASE1**	2	1	Cancer, Organismal Injury and Abnormalities, Respiratory Disease
6	**CBLN4**, DCC, NTN1	2	1	Cell Morphology, Cellular Assembly and Organization, Cellular Development
7	CLOCK, **SCN4B**, SCN5A	2	1	Developmental Disorder, Neurological Disease, Organismal Survival
8	APP, **CST3**, CTSB, CTSD, MMP25	2	1	Cardiovascular Disease, Cellular Assembly and Organization, Developmental Disorder
9	CUL2, F2, GZMA, HABP2, SDC1, **SERPINC1**, USP2	2	1	Cardiovascular Disease, Hematological Disease, Hereditary Disorder

The networks were identified using IPA computational algorithms consisting of the 50 selected proteins (Table 2) and their direct interactions with other proteins (“interconnecting”) in the knowledge base. Scores were calculated for each network according to its fit to the set of selected proteins and used to rank networks on the Ingenuity analysis (version 73,620,684 Release: 2022-03-12). The scores take into account the number of selected proteins and the size of the network to approximate the relevance of the network to the original list of selected proteins. Selected molecules are in bold font (see Table 2) and interconnecting proteins identified in the network are in normal font. See also Tables S6 and S7.

The proteins among the 50 selected included in the networks have peripheral position except for PRKN (parkin) which is indirectly related to NPPB (natriuretic peptides), B2M (beta-2-microglobulin), and KAT2B (histone acetyltransferase KAT2B) (Figure 4A). The GO enrichment analysis selected two significant clusters “regulation of protein binding” (GO ID: 0,043,393) and “regulation of peptidase activity” (GO ID: 0,052,547). The first cluster identified five proteins with two present in network 1 (PRKN, and B2M) and two present in network 2 (WNT3A and ADIPOQ) (Figure 4B). The second cluster identified seven proteins with three present in network 2 (WNT3A, SERPINA4 and ITIH2), two in network 1 (KNG1 and SERPINA7) and one (CST3) in network 8 and, one (SERPINC1) in network 9 (Table S6).

We also examined the significant relationship with the biological pathways involved in “Function and Diseases pathways” (ranked below 500 in IPA) related to “cardiovascular disease” and “cardiovascular system development and function” (Table S7). The key proteins were in the high-scoring networks as well in networks 8 and 9 with NPPB and DPP4 (dipeptidylpeptidase 4) (network 1), PDE4A (phosphodiesterase 4A) and ADIPOQ (adiponectin) (network 2), CST3 (Cystatin-C) (network 8) and SERPINC1 (antithrombin-III) (network 9) present in most cardiovascular diseases selected. All these proteins were associated with hypertension for which we found significant differences between the two groups of variables identified in both cohorts (Table 3).

## Discussion

This study investigated the ability of a large set of biomarkers to identify post-MI patients with long-term occurrence of hospitalization for HF. Our results showed that a subset of 50 plasma proteins measured at time of hospitalization allowed identifying two groups of patients with distinct proteomic expression profiles associated with different occurrence of HF hospitalization. Network analysis identified pathways up-regulated in MI patients with high risk of long-term adverse outcome related to cell-to-cell signaling and cardiovascular disease.

### Protein signature for high risk of adverse outcome after in patients with MI

The relationships between individual biomarkers and adverse outcome following MI have been previously reported. Increased levels of BNP, cardiac troponin, and C-reactive protein were associated with major cardiac events after MI.^8^^,^^9^^,^^10^ In more recent studies, matrix metalloproteinases and other biomarkers of extracellular matrix turnover have also been shown to predict outcome in this setting.^11^^,^^12^ For decades, risk prediction in clinical practice has been based on generally available clinical characteristics and conventional cardiac biomarkers such as BNP.

The present study is one of the first to assess the relationship between a large set of biomarkers (>4,500 proteins) and long-term clinical outcome using high-throughput technology combined with state-of-art statistical and clustering analyses. We used two prospective cohorts, REVE-1 and REVE-2, which included patients suffering from a first acute MI with blood sampling during hospitalization. Patients underwent long-term follow-up with nearly no loss of patients. Thanks to the SOMAscan assay (version 4.0), 4,668 proteins were measured in the plasma of all the patients included in both cohorts. These proteins were not targeted toward any particular disease allowing us to study the link between the event and the proteins without preconceived idea of which proteins should be investigated thus leading to the potential discovery of new biomarkers.

The high-dimensional data generated by these two studies, with more variables than individuals, makes standard statistical analysis unusable. To minimize the risk of overfitting, state of the art statistical approaches with rigorous stability selection procedure were used to ensure reliability of our finding. First, we selected 50 proteins over the large panels of proteins that were significantly associated with the occurrence of HF. Second, a clustering algorithm was used on these 50 proteins giving the same weight to all of them, resulting in two groups where the information of all the proteins is equally represented. Third, competing risk models were then used to model the occurrence of hospitalization for HF against death for all causes using the group variable: low and high risk of occurrence of HF. To validate these effects of groups, we used the validation cohort, REVE-2, for which standardization parameters were set up from the data of the derivation cohort, REVE-1, enabling to use them for other cohorts as we did with REVE-2. We confirmed the effect of groups on occurrence of HF characterized in REVE-1 using competing risk models in REVE-2, as in REVE-1. These two groups of patients only identified by their circulating levels of proteins also showed differences in the clinical characteristics allowing the stratification of the patients in two groups, low and high risk of adverse outcome following MI. These results may help clinicians for more targeted and personalized treatments for patients regarding their cardiac outcome.

The two identified groups of this study based on the measurement of 50 selected proteins with the same technology can easily be applied to other cohorts (or single patient) using the standardization parameters set up.

### Translation of the identified proteins into biological pathways

The proteins found in our network analysis were translated into biological pathways typically related to HF. The network analysis showed that pathways specifically upregulated in MI patients with high risk of hospitalization for HF (group 2) were related to cell-to-cell communication and cardiovascular disease.

The key proteins are NT-proBNP, ADIPOQ, SERPINC1, and WNT3A. NT-proBNP is associated with cardiac stretch with plasma levels widely used for screening and diagnosis of HF^13^ and was previously found to be a specific hub in network analysis of patients with HF and reduced ejection fraction in two independent studies.^14^^,^^15^ ADIPOQ has been shown to affect the autophagic response in the heart and contribute to accelerate cardiac remodeling.^16^ NT-proBNP was closely related in network 1 with PRKN, protein involved in autophagy and both were represented in most cardiovascular diseases (Table S7). SERPINC1 was also highly represented in cardiovascular diseases and by GO enrichment, but its potential as biomarker in HF has not been described up to now. Same for WNT3A, which is found in the 2 selected clusters by GO analysis and has been shown to be involved in cardiac muscle cell differentiation and its upregulation has been shown to be involved in TGFβ1-induced cardiac hypertrophy^17^ but also in cardiomyocyte injury following hypoxia.^18^

### Clinical implications and future perspectives

Individualized risk assessment is an integral part of management for patients in the post-MI setting. Early determination of the 50 selected proteins may help to detect patients at high risk of adverse outcome in the post-MI period. Such identification may encourage more aggressive therapy for this high-risk group. The identified groups were not impacted by the clinical differences between the two cohorts, showing the robustness of the two groups to clinical variations. Regarding this, the group’s ability to predict HF for patients from other cohorts should remain relevant. Finally, the pathophysiological mechanisms beyond the selected proteins enriched in the networks that are highly represented in cardiovascular diseases remains to be established.

### Limitations of the study

The mean sampling time of the two cohorts are different but the results are still significant, which shows that the identified groups are robust to sampling time variations and could be used for clinical prognosis.

The choice of the silhouette criteria for the number of groups could be discussed as many criteria exist in the literature. We believe that two groups are appropriate and lessen the risk of overfitting. Although we believe our strategy valid, external validation in large cohorts from other areas/countries is mandatory to confirm the predictive value of the identified biomarkers subset to classify the MI patients in high- or low-risk of adverse outcome. Our clustering approach was validated in REVE-2, the validation cohort. The patients from the validation cohort were recruited in the same region as the derivation cohort, but standardization parameters were set up for an external validation with other cohorts of patients recruited everywhere in which circulating proteins were measured by a similar technology.

Although most patients had acute reperfusion, the proportion of patients with primary PCI reflects the practice in 2002–2004 and 2006–2008 and was lower than it would be nowadays. Finally, our study populations consisted of mainly men (74 and 81%, respectively in REVE-1 and REVE-2), therefore our prediction model may be less suitable for women. In addition, the patients recruited in the two studies suffered from severe MI and the results might not apply to the overall population of patients after MI.

### Conclusions

Here, we have investigated the cardiac prognostic implications of the highest panel of biomarkers available in MI patients, thanks to the aptamer-based platform. This study has several clinical implications. First, we improve the prediction of the long-term risk of HF occurrence in MI patients; second, the results obtained provide biological context for long-term adverse cardiac outcomes. The proteins found in our network analysis were in link with cardiovascular diseases.

We were able to validate our findings in an independent cohort, significantly reducing the overfitting effect by focusing on proteins linked to the outcome to ensure that results are specific to HF. These promising findings will require external validation in additional ethnic groups of patients.

## STAR★Methods

### Key resources table

**Table:** 

REAGENT or RESOURCE	SOURCE	IDENTIFIER
**Biological samples**		

Plasma samples from REVE-1 study (n=254 patients)	REVE-1 study CHU deLille, FRANCE	https://doi.org/10.1016/j.amjcard.2006.06.011. Epub 2006 Aug 31.
Plasma samples from REVE-2 study (n=238 patients)	REVE-2 study CHU deLille, FRANCE	https://doi.org/10.1016/j.amjcard.2010.06.071. Epub 2010 Oct 1.

**Critical commercial assays**		

BNP measurement by 2-site sandwich assay	Siemens Diagnostic, Zurich, Switzerland	Advia Centaur XPT

**Deposited data**		

Somascan technology Version 4.0	This paper, note 1	See Tables 2 andS4

**Software and algorithms**		

Ingenuity Pathways analysis	Ingenuity Systems	Version: 73620684, Release: 2022-03-12
R	This paper	Version 4.0.2

### Resource availability

#### Lead contact

Further information and requests for resources and reagents should be directed to and will be fulfilled by the lead contact, Florence Pinet (florence.pinet@pasteur-lille.fr).

#### Materials availability

This study did not generate new unique reagents.

There are restrictions to the availability of SOMAScan (property of Somalogic company) and human samples (French cohorts of patients associated with clinical information).

### Method details

#### Data deposition and materials sharing

The data and methods used in the analysis are available to any researcher for purposes of reproducing the results or replicating the procedures.

#### Study populations

The REVE studies have been previously reported.^5^^,^^6^ REVE-1 (inclusion period, February 2002-June 2004, n=266 patients) was designed to test the hypothesis that genetic polymorphisms in candidate genes may be associated with left ventricular remodeling.^7^ REVE-2 (inclusion period, February 2006-September 2008, n=246 patients) was designed to analyze the association of circulating biomarkers with left ventricular remodeling.^6^ Both studies were prospective with a multicentric recruitment. The research protocol of both studies was approved by the ethics committee of the “Centre Hospitalier et Universitaire deLille” (Lille, France), and written informed consent was obtained from each patient. Requalification of samples for SOMAscan analysis was obtained by “Comité deProtection des personnes Nord Ouest IV” (February 2018). IRB approval was obtained and that subjects gave informed consent. The inclusion criteria were the same: a first anterior Q-wave MI with ≥ 3 akinetic segments at predischarge echocardiography. Exclusion criteria were inadequate echocardiography image quality, life-limiting noncardiac disease, significant valvular disease, or previous Q-wave MI in both studies. A long-term clinical follow-up was performed by contacting the general practitioner or cardiologist, or the patients themselves.^7^ We collected data on death and hospitalization for HF. All events occurring during follow-up were adjudicated by two investigators with a third opinion in cases of disagreement. For hospitalizations during the follow-up period, hospital records were reviewed for evidence of clinical events. The events reported by the patients were systematically confirmed from the medical records. Hospitalization for HF was defined as hospitalization for symptoms of dyspnea or edema, elevated venous pressure, or interstitial or alveolar edema on chest X-ray, or the addition of intravenous diuretics or inotropic medications. The primary endpoint of the present study was hospitalization for HF and the competing event was death from all causes.

##### Plasma proteomics measurements

Plasma protein levels were measured in both cohorts with SOMAscan technology (version V4·0).^19^^,^^20^^,^^21^ Peripheral blood samples were collected in EDTA-treated tubes for 255 patients in REVE-1 and 238 patients in REVE-2 after MI during the initial hospitalization. The mean blood sampling day was 7·4 ± 3·9 days and 4·1 ± 2·2 after MI, respectively in REVE-1 and REVE-2. Blood samples were then assayed according to the manufacturer’s protocol, as previously described with the 1·3k assay.^22^ Protein levels from the SOMAScan assay are expressed as relative fluorescence unit (RFU).

### Quantification and statistical analysis

#### Standardization of proteomic measurement

There were no missing values in the set of the 5284 proteins quantified in both cohorts. A total of 414 SOMAmer measurements were removed from the analysis on request of the manufacturer. In the data set, 197 identical proteins were measured using two to three SOMAmers (Table S1). In order to give the same weight to each protein in the data set, for the proteins measured by several SOMAmers, the mean was calculated leading to 4668 proteins ready for analysis.

Proteomic expression levels were log2 transformed. Data quality was checked, and no variation was found between the 15 plates used for the measurements (Figure S1). Results of brain natriuretic peptide (BNP) measurements with the SOMAScan assay were also compared to BNP measurements performed with the automated 2-site sandwich immunoassay on an Advia Centaur (Siemens Diagnostic, Zurich, Switzerland) and showed high correlation (r=0·992). Log2 transformed protein expression levels were standardized (centered and reduced) for each protein on REVE-1, the derivation cohort. These standardization parameters (mean and standard deviation) used in REVE-1 (Table S2) were then applied in REVE-2, the validation cohort.

#### Imputation of missing clinical values

Over the 27 clinical variables studied corresponding to 6858 and 6426 data of variables, respectively for all the patients from REVE-1 and REVE-2, 53 and 69 individual clinical data were missing respectively in REVE-1 (corresponding to 0·0077%) and REVE-2 (corresponding to 0·0107%). These values were imputed using the single imputation method missMDA.^23^

Figure 1 shows the strategy used for analyzing the data obtained in the derivation (REVE-1) and validation (REVE-2) cohorts.

#### Proteins selection

In order to only focus on the relevant proteins, with a significant effect on the occurrence of hospitalization for HF, univariate competing risk models were fitted for each protein individually using R package cmprsk (version 2·2-11).^24^ For each protein, the occurrence of hospitalization for HF was modeled in competition with the occurrence of all-causes death using the competing risk model as previously defined.^25^ Tests were performed on the subhazard ratio (SHR) to measure the significance of the relationship between the protein’s expression and the occurrence of hospitalization for HF. Then, in order to take into account multiple testing, the significance threshold was set to 1·07 10^-5^, which corresponds to the FamilyWise Error Rate (FWER) at 0.05 with the Bonferroni method^26^ for 4668 tests. The proteins significantly associated with the event in REVE-1 were selected for the following analyses.

#### Clustering of patients based on selected proteins

A *k*-means clustering algorithm^27^ was applied in the REVE-1 study using the selected proteins as variables in order to define groups of patients. This algorithm builds groups of individuals sharing similar values for protein expressions regardless of the risk of hospitalization for HF of the patients. Individuals are gathered into groups characterized by their centers and each patient is assigned to the group whose center is the closest in terms of Euclidean distance. Therefore, a new categorical variable called “group variable” was defined with these affectations for the following analyses.

In order to choose a suitable number of groups, the overall average silhouette width was computed.^28^ The silhouette refers to a method of interpretation and validation of consistency within clusters of data. The average silhouette width is calculated with Euclidean distance to measure how similar is the mean distance of each individual compared to other individuals in the same group and to the individuals from other groups. The silhouette ranges from –1 to +1, where the closest value to 1 indicates that the clustering is appropriate. This criterion was computed for a number of groups varying from 2 to 6. Finally, the number of groups which gave the higher overall average silhouette width was used.

As data were standardized using the same parameters for both cohorts, patients of REVE-2 were then assigned to the previously built groups in REVE-1 using the same method.

Group differences were identified using Welch’s test of mean equality for quantitative variables (both for clinical data and for differential proteomic analysis) or chi squared test for clinical categorical variables. Differences corresponding to raw p-values below 0.05 were considered significant.

#### Groups’effect on the occurrence of HF

In order to assess the interest of the previously created groups, cumulative incidence curves of hospitalization for HF were drawn for both groups. For each cohort, competing risk models were then used to model the occurrence of hospitalization for HF using the group variable. This allows to measure the strength and to test the significance of the group effect. Models were fitted for both cohorts in order to measure the effect of the groups in the derivation and the validation cohort. The models were then adjusted on the clinical variables age, gender, ejection fraction, diabetes, killip class, serum creatinine, BNP and NT-proBNP separately, in order to ensure the robustness of the information provided by the identified groups after adding relevant clinical information.

Sensitivity analysis was performed on the selected proteins without the established cardiac biomarkers (BNP, NT-proBNP). Incidence curve were drawn for the group identified with the selected proteins minus the established biomarkers and SHR competing risk model was fitted.

All statistical analyses were made using R (version 4.0.2).

#### Enrichment analysis

Gene ontology enrichment analysis was performed on the set of selected proteins using Biological Processes, Molecular Function and Cellular Component subsets. Enrichments tests were performed using the R package clusterProfiler.^29^ For each GO term, a Fisher exact test was performed in order to test for the overrepresentation of the set of selected proteins in the GO term.

#### Ingenuity Pathways Analysis

The proteins of interest, selected to be significantly associated with occurrence of HF**,** were subjected to Ingenuity Pathways Analysis (IPA) (Current version: 73620684 Release: 2022-03-12, Ingenuity Systems) and used as a starting point for building biological networks. This analysis uses computational algorithms to identify networks consisting of focus proteins (proteins significantly modulated) and their interactions with other proteins (“non-focused”) in the knowledge base. Scores were calculated for each network according to the fit of the network to the set of focus proteins and used to rank networks on the IPA database restricted to direct interactions. IPA uses the proteins from the highest-scoring network to extract a connectivity pathway that relates candidate proteins to each other based on their direct interactions. IPA determines the “Function & Disease and Canonical Pathways” to be significantly associated with these candidate proteins.

#### SOMAscan V4 data standardization and file specification technical note

##### Overview

Normalization and calibration are routine numerical procedures developed to remove systematic biases in the raw assay data. Normalization is a sample-by-sample adjustment in overall signal within a single plate (run) performed across three non-consecutive steps: Hybridization Control Normalization, Intraplate Median Signal Normalization, and Median Signal Normalization to a Reference. Plate Scaling and Calibration is a SOMAmer® binding reagent-by-SOMAmer binding reagent adjustment that minimizes between-plate variability. Global reference standards are established for procedures with controls on each plate individual, QC, and Calibrator samples are normalized and calibrated to the established global reference standards. Separate calibrator global reference standards are established for each matrix (serum, plasma), and assay shifts or skew from the global reference standards are tracked over time. New global reference standards may be developed in concordance with changes in assay processes, performance, or reagents.

Hybridization Control Normalization was developed to remove systematic biases present in the raw data after slide feature aggregation from a slide-based hybridization microarray for assay readout and quantification. Hybridization Control Normalization is performed using a set of twelve hybridization control sequences measured independently for each sample array. The procedure is intended to correct for systematic effects on the data introduced during the hybridization readout and results in a single scale factor for each sample that is subsequently applied to the measured signal on all features within a subarray (sample).

Intraplate Median Signal Normalization uses all the SOMAmer reagent signals on a given subarray to remove sample or assay biases that may be due to differences between samples in overall protein concentration, pipetting variation, variation in reagent concentrations, assay timing, and any other source of systematic variability within a single plate. Each SOMAmer reagent is assigned to one of three dilution sets, scale factors are derived within dilution sets separately, and all SOMAmer binding reagents within each set are scaled together. Three sample dilutions will result in three independent median signal scale factors for each subarray (sample) in addition to the hybridization scale factor. Thisstep is only applied to calibrator samples.

Plate Scaling and Calibration is accomplished using a number of replicate measurements of a common pooled calibrator sample consistent with the assay sample type for a study. Calibrator samples must be composed of identical sample matrices as the samples that are being calibrated. No protein spikes are added to the calibrator samples – SomaLogic relies solely on the endogenous levels of each analyte within a calibrator sample. Since calibration attempts to correct plate-to-plate variation and such variation can be idiosyncratic for SOMAmer binding reagents, a unique calibration scale factor is derived for each SOMAmer binding reagent within the assay. The median of these scale factors is then computed and applied across all SOMAmer measurements in that plate to account for the total signal difference (plate scale), and the scale factors are subsequently recalculated for each SOMAmer and applied to all measurements within the set of samples in that plate.

Median Signal Normalization to a Reference occurs on a per-sample basis, wherein a scale factor for a set of SOMAmer reagents is computed against a reference value generated from a cohort of healthynormal individuals and then aggregated within a dilution. The median of each dilution’s scale factors is then applied to their respective SOMAmer reagents. Thisstep is applied to QC, Buffer, and individual samples.

File specification: SOMAscan results are produced in a tab-delimited ASCII file with an ADAT extension (ADAT file). The ADAT file contains measurements for a series of analytes (columns) across a series of samples (rows) and includes analyte description and sample description information. The format is designed to provide flexibility for the number of samples as well as the number and types of analyte and sample descriptors.

##### Normalization

###### Hybridization Control Normalization

A set of hybridization control sequences is added to each sample as part of the elution buffer in the SOMAscan assay. These hybridization controls are added at concentrations to give measured relative fluorescence units (RFU) that span the dynamic range of the assay. The global reference RFU value for each hybridization control is defined by the median signal measured within the current plate being normalized. A ratio is computed by dividing the median RFU for each control by its measured RFU in the sample. The median of these hybridization control measurement ratios in each subarray defines the sample-based hybridization scale factor. By definition, such a scaling will equate the median RFU for the hybridization controls to the median reference RFU for the controls. All SOMAmer reagent results within a sample are multiplied by the resulting hybridization scale factor increasing or decreasing the overall “brightness” of the sample. The procedure is displayed graphically in Note Figure S4.

###### Intraplate Median Signal Normalization

Intraplate Median Signal Normalization is performed on each sample dilution independently. In most matrices, each SOMAmer binding reagent is assigned to one of three dilution sets, scale factors are derived within dilution sets separately, and all SOMAmer reagents within each set are scaled together. Within each sample matrix, this is only performed on calibrator samples. Like Hybridization Control Normalizationwhich uses a local reference standard, the local median reference RFU for each SOMAmer reagent is the median RFU for that SOMAmer binding reagent within the sample group (calibrator in buffer) in the plate to be normalized. As in hybridization normalization, a ratio is computed for each SOMAmer reagent by dividing the reference SOMAmer RFU by its measured RFU in the sample to be normalized. The median of the SOMAmer measurement ratios for all SOMAmer reagents in a dilution defines the sample-based scale factor for all SOMAmer reagents within that dilution and sample. All SOMAmer reagents within the dilution for a sample are scaled by the resulting median signal scale factor. Three sample dilutions will result in three independent median signal scale factors for each sample in addition to the hybridization scale factor as shown in Note Figure S5.

##### Plate Scaling &calibration

Clinical sample studies are plate scaled and calibrated to remove systematic assay variability. A set of control calibrator samples is used to detect and remove systematic variability between independent assay plates. Calibrator samples must be of the same type as the samples that are being calibrated. Calibrator global reference RFU values for each SOMAmer reagent are defined by the median signal measured on a set of samples spanning a number of independent assay plates that have been shown to meet assay acceptance criteria. For each SOMAmer reagent, the median RFU signal for that SOMAmer reagent across all the calibrator samples within the clinical study defines the global calibrator reference for that SOMAmer binding reagent. Note Figure S6 below displays the data from a typical clinical study and illustrates the systematic bias removed by calibration.

Plate scaling is performed on an entire independent plate. A local median reference value is derived for each SOMAmer reagent by computing the median RFU for that SOMAmer reagent from the set of replicate calibrator samples within the plate. The SOMAmer-based calibration scale factor is then computed by dividing the calibrator global reference RFU by the local median reference value defined for each SOMAmer reagent. The median of all scale factors for a given plate is then applied across all SOMAmer measures in the plate (plate) forcing the overall calibrator median signal to match the overall median signal within the global calibrator reference.

Plate-to-plate calibration is performed on each SOMAmer measurement within the plate independently. A local median reference value is derived for each SOMAmer reagent by computing the median RFU for that SOMAmer reagent from the set of replicate calibrator samples within the plate. The SOMAmer-based calibration scale factor is then computed by dividing the calibrator global reference RFU by the local median reference value defined for each SOMAmer reagent. This scale factor is applied to all SOMAmer measurements in the plate, forcing the median calibrator signal to match the global calibrator reference for that SOMAmer binding reagent. Each plate within a study has a unique calibration scale factor for each SOMAmer reagent. The data from Note Figure S5 are displayed after calibration in Note Figure S6.

##### Median Normalization to a reference

All individual, QC, and Buffer samples are then median normalized to a reference value. Unlike Intraplate Median Signal Normalization, Median Normalization to a Reference can be performed on a single sample due to the presence of an external global reference value generated from a cohort of healthynormal individuals for each SOMAmer reagent. This method is very similar to Intraplate Median Signal Normalization in practice, the primary difference being the origination of the reference value. A ratio is computed for each SOMAmer reagent by dividing the global reference SOMAmer RFU by its measured RFU in the sample to be normalized. The median of the SOMAmer measurement ratios for all SOMAmer reagents in a dilution defines the sample-based scale factor for all SOMAmer reagents within that dilution and sample. All SOMAmer reagents within the dilution for a sample are scaled by the resulting median signal scale factor. Three sample dilutions will result in three independent median signal scale factors for each sample in addition to the hybridization scale factor.

##### Acceptance criteria

Hybridization Control and Intraplate Median Signal Normalization scale factors are expected to be in the range of 0.4–2.5. The plate scale factor is expected to be between 0.4 and 2.5. The distribution of QC sample ratios is expected to have 85% of individual SOMAmer reagents in the total array between 0.84 and 1.19 (i.e. less than 15% in the tails of the distribution). Gaussian distributions of scale factors are expected. A report is provided for each study (single plate or set of plates) with the results of the Normalization and Calibration process.

## Data Availability

Any additional information required to reanalyze the data reported in this paper is available from the lead contact upon request. Data: All (clinical and proteomic) data reported in this paper will be shared by the lead contact upon request. Code: This paper does not report original code.

## Supplemental information

Document S1. Tables S2–S6 and Figures S1–S6

Table S1. List of the proteins quantified by several SOMAmers, related to Table 2

Table S7. Diseases and functions in REVE-1 network, related to Table 4 and Figure 4
