# Supplementary material for: Identification of patient subtypes based on protein expression for prediction of heart failure after myocardial infarction
Source: iScience. 2023 Feb 11;26(3):106171. doi: 10.1016/j.isci.2023.106171 (PMC10006628; doi:10.1016/j.isci.2023.106171)
Supplement: Document S1. Tables S2–S6 and Figures S1–S6 [file mmc1.pdf]

**iScience, Volume *26***

## Supplemental information

# Identification of patient subtypes based on protein expression for prediction of heart failure after myocardial infarction

**Wilfried Heyse, Vincent Vandewalle, Guillemette Marot, Philippe Amouyel, Christophe Bauters, and Florence Pinet**

**Table S2. Standardization parameters of the selected proteins computed on REVE-1, related to Table 2.**

| UniProt ID | Symbol | Mean | Standard Deviation |
|---|---|---|---|
| C9JXX5 | C11ORF94 | 9·23 | 0·57 |
| P27815 | PDE4A | 7·57 | 0·44 |
| Q9Y3Z3 | SAMHD1 | 9·86 | 0·46 |
| Q9Y644 | RFNG | 10·64 | 0·42 |
| O75473 | LGR5 | 8·99 | 0·31 |
| Q16568 | CARTPT | 8·30 | 0·56 |
| P56704 | WNT3A | 11·52 | 0·5 |
| P05543 | SERPINA7 | 15·84 | 0·25 |
| P19823 | ITIH2 | 16·20 | 0·26 |
| Q8TAT2 | FGFBP3 | 10·20 | 0·56 |
| Q92831 | KAT2B | 8·83 | 0·55 |
| Q9Y251 | HPSE | 9·77 | 1·01 |
| A8K7I4 | CLCA1 | 9·34 | 0·66 |
| Q9NTU7 | CBLN4 | 10·37 | 0·46 |
| P61769 | B2M | 10·09 | 0·38 |
| Q2I0M5 | RSPO4 | 8·83 | 0·34 |
| P52943 | CRIP2 | 10·13 | 0·38 |
| P33764 | S100A3 | 8·64 | 0·27 |
| *Q9BUP3* | *HTATIP2* | *10·78* | *0·36* |
| Q96PQ0 | SORCS2 | 10·14 | 0·82 |
| Q8IWT1 | SCN4B | 9·15 | 0·41 |
| Q6P988 | NOTUM | 10·05 | 0·46 |
| P29622 | SERPINA4 | 15·50 | 0·27 |
| P01034 | CST3 | 11·26 | 0·38 |
| Q8N6C8 | LILRA3 | 10·85 | 0·88 |
| Q9UNP4 | ST3GAL5 | 8·06 | 0·42 |
| Q8N6M8 | IQCF1 | 7·97 | 0·39 |
| P0DJ93 | SMIM13 | 9·65 | 0·35 |
| P01008 | SERPINC1 | 16·40 | 0·19 |
| Q8TE54 | SLC26A7 | 10·49 | 0·49 |
| Q6ZUB0 | SPATA31D4 | 8·54 | 0·26 |
| P16860 | NPPB | 8·78 | 0·69 |
| Q12805 | EFEMP1 | 9·41 | 0·45 |
| Q5H8A3 | NMS | 5·99 | 0·26 |
| P35858 | IGFALS | 14·45 | 0·46 |
| Q9NS68 | TNFRSF19 | 9·48 | 0·46 |
| O60260 | PARK2 | 10·15 | 0·36 |
| Q13522 | PPP1R1A | 9·90 | 0·48 |
| P55285 | CDH6 | 8·18 | 0·45 |
| P45985 | MAP2K4 | 13·51 | 0·66 |
| Q2Y0W8 | SLC4A8 | 8·37 | 0·24 |
| P01042 | KNG1 | 10·89 | 0·62 |
| Q8N3Z0 | PRSS35 | 8·75 | 0·42 |

| | | | |
|---|---|---|---|
| P27487 | DPP4 | 10·56 | 0·30 |
| Q8IYN2 | TCEAL8 | 8·33 | 0·26 |
| Q15848 | ADIPOQ | 11·25 | 0·71 |
| Q9UII4 | HERC5 | 10·31 | 0·30 |
| P07998 | RNASE1 | 9·78 | 1·05 |
| Q9UBC7 | GALP | 8·75 | 0·31 |
| Q9UK76 | JPT1 | 12·71 | 0·85 |

The standardization step has computed the mean and standard deviation for each protein after log2 transformed data from each patient of REVE-1 cohort. For the proteins quantified by several SOMAmers, indicated in italic, the mean of these SOMAmers was used for the standardization. The table only indicates the computed values of the 50 proteins selected. The proteomic data of REVE-1 and REVE-2 have been standardized protein by protein, using these values following the calculation: (value of the protein - the mean of protein in REVE-1) / the corresponding standard deviation.

**Table S3. Location and type of the 50 plasma proteins selected in Table 2, related to Table 2.**

| UniProt ID | Symbol | Entrez Gene Name | Location | Type(s) |
|---|---|---|---|---|
| C9JXX5 | C11ORF94 | Uncharacterized protein C11orf94 | Extracellular space | Other |
| P27815 | PDE4A | cAMP-specific 3',5'-cyclic phosphodiesterase 4A | Cytoplasm | Enzyme |
| Q9Y3Z3 | SAMHD1 | Deoxynucleoside triphosphate triphosphohydrolase SAMHD1 | Nucleus | Enzyme |
| Q9Y644 | RFNG | Beta-1,3-N-acetylglucosaminyltransferase radical fringe | Cytoplasm | Enzyme |
| O75473 | LGR5 | Leucine-rich repeat-containing G-protein coupled receptor 5 | Plasma membrane | Transmembrane receptor |
| Q16568 | CARTPT | Cocaine- and amphetamine-regulated transcript protein | Extracellular space | Other |
| P56704 | WNT3A | Protein Wnt-3a | Extracellular space | Cytokine |
| P05543 | SERPINA7 | Thyroxine-binding globulin | Extracellular space | Transporter |
| P19823 | ITIH2 | Inter-alpha-trypsin inhibitor heavy chain H2 | Extracellular space | Other |
| Q8TAT2 | FGFBP3 | Fibroblast growth factor-binding protein 3 | Extracellular space | Other |
| Q92831 | KAT2B | Histone acetyltransferase KAT2B | Nucleus | Transcription regulator |
| Q9Y251 | HPSE | Heparanase | Plasma membrane | Enzyme |
| A8K7I4 | CLCA1 | Calcium-activated chloride channel regulator 1 | Plasma membrane | Ion channel |
| Q9NTU7 | CBLN4 | Cerebellin-4 | Extracellular space | Other |
| P61769 | B2M | Beta-2-microglobulin | Plasma membrane | Transmembrane receptor |
| Q2I0M5 | RSPO4 | R-spondin-4 | Plasma membrane | Other |
| P52943 | CRIP2 | Cysteine-rich protein 2 | Nucleus | Other |

| | | | | |
|---|---|---|---|---|
| P33764 | S100A3 | Protein S100-A3 | Nucleus | Transporter |
| *Q9BUP3* | *HTATIP2* | *Oxidoreductase HTATIP2* | *Nucleus* | *Transcription regulator* |
| Q96PQ0 | SORCS2 | VPS10 domain-containing receptor SorCS2 | Plasma membrane | Transporter |
| Q8IWT1 | SCN4B | Sodium channel subunit beta-4 | Plasma membrane | Ion channel |
| Q6P988 | NOTUM | Palmitoleoyl-protein carboxylesterase NOTUM | Extracellular space | Other |
| P29622 | SERPINA4 | Kallistatin | Extracellular space | Other |
| P01034 | CST3 | Cystatin-C | Extracellular space | Other |
| Q8N6C8 | LILRA3 | Leukocyte immunoglobulin-like receptor subfamily A member 3 | Extracellular space | Other |
| Q9UNP4 | ST3GAL5 | Lactosylceramide alpha-2,3-sialyltransferase | Cytoplasm | Enzyme |
| Q8N6M8 | IQCF1 | IQ domain-containing protein F1 | Cytoplasm | Other |
| P0DJ93 | SMIM13 | Small integral membrane protein 13 | Other | Other |
| P01008 | SERPINC1 | Antithrombin-III | Extracellular space | Enzyme |
| Q8TE54 | SLC26A7 | Anion exchange transporter | Plasma Membrane | Transporter |
| Q6ZUB0 | SPATA31D4 | Putative spermatogenesis-associated protein 31D4 | Other | Other |
| P16860 | NPPB | Natriuretic peptides B | Extracellular space | Other |
| Q12805 | EFEMP1 | EGF-containing fibulin-like extracellular matrix protein 1 | Extracellular space | Enzyme |
| Q5H8A3 | NMS | Neuromedin-S | Extracellular space | Other |
| P35858 | IGFALS | Insulin-like growth factor-binding protein complex acid labile subunit | Extracellular space | Other |
| Q9NS68 | TNFRSF19 | Tumor necrosis factor receptor superfamily member 19 | Plasma membrane | Transmembrane receptor |

| | | | | |
|---|---|---|---|---|
| O60260 | PARK2 | E3 ubiquitin-protein ligase parkin | Cytoplasm | Enzyme |
| Q13522 | PPP1R1A | Protein phosphatase 1 regulatory subunit 1A | Cytoplasm | Phosphatase |
| P55285 | CDH6 | Cadherin-6 | Plasma membrane | Other |
| P45985 | MAP2K4 | Dual specificity mitogen-activated protein kinase kinase 4 | Cytoplasm | Kinase |
| Q2Y0W8 | SLC4A8 | Electroneutral sodium bicarbonate exchanger 1 | Plasma membrane | Transporter |
| P01042 | KNG1 | Kininogen, HMW, Two Chain | Extracellular space | Other |
| Q8N3Z0 | PRSS35 | Inactive serine protease 35 | Extracellular space | Peptidase |
| P27487 | DPP4 | Dipeptidyl peptidase 4 | | |
| Q8IYN2 | TCEAL8 | Transcription elongation factor A protein-like 8 | Other | Other |
| Q15848 | ADIPOQ | Adiponectin | Extracellular space | Other |
| Q9UII4 | HERC5 | E3 ISG15--protein ligase HERC5 | Cytoplasm | Enzyme |
| P07998 | RNASE1 | Ribonuclease pancreatic | Extracellular space | Enzyme |
| Q9UBC7 | GALP | Galanin-like peptide | Extracellular space | Other |
| Q9UK76 | JPT1 | Jupiter microtubule associated homolog 1 | Nucleus | Other |

UniProt ID, protein symbol, full protein name are provided by the UniProtKB database (https://www.uniprot.org). Subcellular location was provided by IPA (version 73620684 Release: 2022-03-12). The proteins in italics were quantified by several SOMAmers and the mean was used.

**Table S4. Plasma levels of the 50 selected proteins in the two identified groups in both cohorts, related to Table 2 and Figure 2.**

| | | REVE-1 (Derivation) (n= 254) | | | REVE-2 (Validation) (n=238) | | |
|---|---|---|---|---|---|---|---|
| **UniProt ID** | **Symbol** | **Group 1 (n=160)** | **Group 2 (n=94)** | ***P* value** | **Group 1 (n=190)** | **Group 2 (n=48)** | ***P* value** |
| C9JXX5 | C11ORF94 | 9·16 ± 0·18 | 9·37 ± 0·89 | 2·54e-02 | 9·25 ± 0·24 | 9·33 ± 0·24 | 5·66e-02 |
| P27815 | PDE4A | 7·54 ± 0·17 | 7·61 ± 0·68 | 3·78e-01 | 7·50 ± 0·30 | 7·43 ± 0·16 | 1·95e-02 |
| Q9Y3Z3 | SAMHD1 | 9·89 ± 0·31 | 9·82 ± 0·64 | 3·09e-01 | 9·91 ± 0·40 | 9·82 ± 0·48 | 2·49e-01 |
| Q9Y644 | RFNG | 10·60 ± 0·24 | 10·71 ± 0·61 | 9·94e-02 | 10·52 ± 0·28 | 10·63 ± 0·38 | 7·78e-02 |
| O75473 | LGR5 | 9·14 ± 0·26 | 8·73 ± 0·20 | 2·33e-32 | 9·33 ± 0·33 | 8·92 ± 0·22 | 6·19e-18 |
| Q16568 | CARTPT | 8·29 ± 0·69 | 8·32 ± 0·22 | 6·33e-01 | 8·23 ± 0·35 | 8·31 ± 0·22 | 8·07e-02 |
| P56704 | WNT3A | 11·77 ± 0·37 | 11·11 ± 0·40 | 3·94e-27 | 11·93 ± 0·37 | 11·46 ± 0·59 | 1·62e-06 |
| P05543 | SERPINA7 | 15·90 ± 0·21 | 15·73 ± 0·27 | 2·54e-07 | 15·88 ± 0·19 | 15·74 ± 0·24 | 5·11e-04 |
| P19823 | ITIH2 | 16·27 ± 0·22 | 16·09 ± 0·29 | 7·69e-07 | 16·18 ± 0·23 | 16·00 ± 0·50 | 1·94e-02 |
| Q8TAT2 | FGFBP3 | 10·04 ± 0·43 | 10·47 ± 0·65 | 6·06e-08 | 10·29 ± 0·73 | 11·03 ± 1·20 | 1·33e-04 |
| Q92831 | KAT2B | 8·76 ± 0·62 | 8·95 ± 0·39 | 3·67e-03 | 8·67 ± 0·28 | 8·96 ± 0·30 | 8·2e-08 |
| Q9Y251 | HPSE | 9·37 ± 0·63 | 10·44 ± 1·18 | 2·85e-13 | 9·06 ± 0·49 | 9·31 ± 0·60 | 8·3e-03 |
| A8K7I4 | CLCA1 | 9·25 ± 0·48 | 9·51 ± 0·86 | 7·83e-03 | 9·16 ± 0·62 | 9·22 ± 0·58 | 5·17e-01 |
| Q9NTU7 | CBLN4 | 10·53 ± 0·41 | 10·09 ± 0·42 | 4·09e-14 | 10·75 ± 0·35 | 10·40 ± 0·47 | 5·76e-06 |
| P61769 | B2M | 9·94 ± 0·25 | 10·34 ± 0·43 | 1·32e-13 | 9·96 ± 0·25 | 10·55 ± 0·56 | 3·29e-09 |
| Q2I0M5 | RSPO4 | 8·75 ± 0·32 | 8·98 ± 0·31 | 1·49e-07 | 8·71 ± 0·35 | 9·02 ± 0·32 | 1·15e-07 |
| P52943 | CRIP2 | 10·11 ± 0·42 | 10·17 ± 0·31 | 1·92e-01 | 10·43 ± 0·30 | 10·60 ± 0·31 | 1·04e-03 |
| P33764 | S100A3 | 8·76 ± 0·20 | 8·43 ± 0·23 | 9·1e-23 | 8·77 ± 0·22 | 8·55 ± 0·23 | 7·44e-08 |
| *Q9BUP3* | *HTATIP2* | *10·69 ± 0·29* | *10·93 ± 0·40* | *8·35e-07* | *10·58 ± 0·26* | *10·67 ± 0·34* | *8·29e-02* |
| Q96PQ0 | SORCS2 | 10·38 ± 0·81 | 9·74 ± 0·66 | 4·6e-11 | 10·69 ± 0·78 | 10·12 ± 0·70 | 4·06e-06 |
| Q8IWT1 | SCN4B | 9·32 ± 0·35 | 8·87 ± 0·34 | 1·8e-19 | 9·17 ± 0·40 | 8·73 ± 0·28 | 3·23e-14 |
| Q6P988 | NOTUM | 10·15 ± 0·43 | 9·88 ± 0·47 | 1·23e-05 | 10·06 ± 0·49 | 9·59 ± 0·57 | 1·94e-06 |
| P29622 | SERPINA4 | 15·59 ± 0·22 | 15·34 ± 0·28 | 1·6e-11 | 15·51 ± 0·23 | 15·31 ± 0·32 | 1·16e-04 |
| P01034 | CST3 | 11·11 ± 0·25 | 11·50 ± 0·44 | 8·14e-13 | 11·17 ± 0·26 | 11·70 ± 0·46 | 1·57e-10 |
| Q8N6C8 | LILRA3 | 10·72 ± 0·99 | 11·05 ± 0·62 | 1·39e-03 | 10·78 ± 0·76 | 10·57 ± 1·46 | 3·4e-01 |
| Q9UNP4 | ST3GAL5 | 8·16 ± 0·48 | 7·89 ± 0·22 | 3·91e-09 | 8·20 ± 0·67 | 8·06 ± 0·71 | 2·01e-01 |

| | | | | | | | |
|---|---|---|---|---|---|---|---|
| Q8N6M8 | IQCF1 | 8·14 ± 0·26 | 7·68 ± 0·41 | 2·83e-17 | 8·27 ± 0·29 | 7·78 ± 0·25 | 8·05e-19 |
| P0DJ93 | SMIM13 | 9·60 ± 0·18 | 9·73 ± 0·53 | 2·16e-02 | 9·64 ± 0·21 | 9·74 ± 0·24 | 1·37e-02 |
| P01008 | SERPINC1 | 16·46 ± 0·17 | 16·31 ± 0·19 | 5·03e-09 | 16·45 ± 0·18 | 16·28 ± 0·45 | 1·06e-02 |
| Q8TE54 | SLC26A7 | 10·33 ± 0·41 | 10·77 ± 0·49 | 6·74e-12 | 10·14 ± 0·31 | 10·43 ± 0·30 | 1·21e-07 |
| Q6ZUB0 | SPATA31D4 | 8·62 ± 0·27 | 8·4 ± 0·17 | 4·16e-13 | 8·65 ± 0·41 | 8·50 ± 0·28 | 3·29e-03 |
| P16860 | NPPB | 8·60 ± 0·56 | 9·09 ± 0·78 | 2·8e-07 | 8·70 ± 0·51 | 9·40 ± 0·72 | 3·08e-08 |
| Q12805 | EFEMP1 | 9·30 ± 0·42 | 9·60 ± 0·45 | 7·2e-07 | 9·33 ± 0·36 | 9·80 ± 0·37 | 2·62e-11 |
| Q5H8A3 | NMS | 5·99 ± 0·20 | 5·99 ± 0·33 | 8·52e-01 | 5·99 ± 0·47 | 6·00 ± 0·45 | 9·61e-01 |
| P35858 | IGFALS | 14·59 ± 0·39 | 14·21 ± 0·47 | 3·68e-10 | 14·60 ± 0·40 | 14·14 ± 0·61 | 8·63e-06 |
| Q9NS68 | TNFRSF19 | 9·39 ± 0·35 | 9·63 ± 0·57 | 5·2e-04 | 9·53 ± 0·35 | 9·90 ± 0·59 | 8·43e-05 |
| O60260 | PARK2 | 10·33 ± 0·31 | 9·86 ± 0·21 | 2·51e-34 | 10·46 ± 0·33 | 10·14 ± 0·29 | 3·69e-09 |
| Q13522 | PPP1R1A | 9·87 ± 0·50 | 9·95 ± 0·43 | 1·75e-01 | 10·30 ± 0·36 | 10·61 ± 0·42 | 1·75e-05 |
| P55285 | CDH6 | 8·38 ± 0·38 | 7·82 ± 0·33 | 6·79e-27 | 8·48 ± 0·41 | 8·00 ± 0·37 | 1·68e-11 |
| P45985 | MAP2K4 | 13·74 ± 0·57 | 13·11 ± 0·62 | 7·04e-14 | 13·79 ± 0·53 | 13·43 ± 0·50 | 3·79e-05 |
| Q2Y0W8 | SLC4A8 | 8·48 ± 0·17 | 8·17 ± 0·19 | 2·25e-28 | 8·62 ± 0·32 | 8·31 ± 0·21 | 8·24e-13 |
| P01042 | KNG1 | 10·99 ± 0·63 | 10·71 ± 0·57 | 2·68e-04 | 11·31 ± 0·64 | 11·00 ± 0·63 | 3·6e-03 |
| Q8N3Z0 | PRSS35 | 8·9 ± 0·39 | 8·49 ± 0·34 | 5·39e-16 | 8·77 ± 0·23 | 8·46 ± 0·28 | 1·82e-09 |
| P27487 | DPP4 | 10·69 ± 0·26 | 10·35 ± 0·23 | 6·5e-22 | 10·58 ± 0·36 | 10·41 ± 0·37 | 5·12e-03 |
| Q8IYN2 | TCEAL8 | 8·43 ± 0·23 | 8·15 ± 0·20 | 1·05e-19 | 8·63 ± 0·34 | 8·39 ± 0·25 | 3·79e-07 |
| Q15848 | ADIPOQ | 11·11 ± 0·69 | 11·50 ± 0·68 | 1·87e-05 | 11·01 ± 0·61 | 11·70 ± 0·63 | 1·83e-09 |
| Q9UII4 | HERC5 | 10·42 ± 0·27 | 10·12 ± 0·27 | 2·55e-15 | 10·22 ± 0·28 | 9·99 ± 0·27 | 1·77e-06 |
| P07998 | RNASE1 | 9·53 ± 0·79 | 10·21 ± 1·27 | 7·48e-06 | 9·84 ± 0·62 | 10·89 ± 1·25 | 6·57e-07 |
| Q9UBC7 | GALP | 8·86 ± 0·32 | 8·58 ± 0·18 | 3·98e-17 | 8·95 ± 0·23 | 8·70 ± 0·20 | 7·02e-11 |
| Q9UK76 | JPT1 | 12·99 ± 0·82 | 12·22 ± 0·69 | 5·88e-14 | 13·03 ± 0·76 | 12·53 ± 0·58 | 2·82e-06 |

Data are log2-transformed and expressed as mean ± standard deviation. The proteins in italics were quantified by several SOMAmers.
*P* values were calculated using a student test in order to test for equality of the proteins expression between the two identified groups.

**Table S5. Subhazard ratios and their *P* values for the group as variable in adjusted models, related to Table 2 and Figure 3.**

| Variables | REVE-1 | | REVE-2 | |
|---|---|---|---|---|
| | SHR | *P* value | SHR | *P* value |
| Group (alone) | 7·26 [3·74 - 14·07] | 4·50e-09 | 3·66 [1·75 - 7·64] | 5·70e-04 |
| Group + Age | 5·59 [2·90 - 10·79] | 2·90e-07 | 2·48 [1·10 - 5·55] | 2·80e-02 |
| Group + Gender | 7·40 [3·83 - 14·30] | 2·60e-09 | 3·49 [1·68 - 7·22] | 7·70e-04 |
| Group + Diabetes | 6·90 [3·56 - 13·36] | 1·00e-08 | 3·21 [1·41 - 7·28] | 5·30e-03 |
| Group + Ejection Fraction | 5·66 [2·79 - 11·49] | 1·60e-06 | 3·53 [1·68 - 7·43] | 8·90e-04 |
| Group + Kilipp Score | 6·63 [3·44 - 12·81] | 1·70e-08 | 3·40 [1·61 - 7·15] | 1·30e-03 |
| Group + Serum Creatinine | 7·28 [3·76 - 14·09] | 3·70e-09 | 3·64 [1·73 - 7·64] | 6·40e-04 |
| Group + BNP | 5·76 [2·95 - 11·27] | 3·00e-07 | 2·56 [1·14 - 5·75] | 2·30e-02 |
| Group + NT-proBNP | 5·88 [3·02 - 11·45] | 1·90e-07 | 2·48 [1·14 - 5·40] | 2·20e-02 |
| Group + Age + Gender | 5·72 [2·99 - 10·92] | 1·30e-07 | 2·45 [1·11 - 5·41] | 2·70e-02 |
| Group + Age + Diabetes | 5·33 [2·79 - 10·19] | 4·10e-07 | 2·29 [1·10 - 5·16] | 4·20e-02 |
| **Group + Age + Ejection Fraction** | **4·50 [2·19 - 9·26]** | **4·30e-05** | **2·19 [1·05 - 4·93]** | **4·70e-02** |
| Group + Age + Kilipp Score | 5·29 [2·76 - 10·15] | 5·50e-07 | 2·19 [1·05 - 4·98] | 4·70e-02 |
| Group + Age + Serum Creatinine | 5·75 [2·98 - 11·12] | 1·90e-07 | 2·48 [1·10 - 5·58] | 2·90e-02 |
| Group + Age + BNP | 5·08 [2·59 - 9·95] | 2·20e-06 | 2·18 [1·06 - 4·85] | 4·50e-02 |
| Group + Age + NT-proBNP | 5·21 [2·67 - 10·16] | 1·30e-06 | 2·19 [1·09 - 4·79] | 4·40e-02 |

| | | | | |
|---|---|---|---|---|
| Group + Gender + Diabetes | 7·09 [3·67 - 13·68] | 5·40e-09 | 2·96 [1·32 - 6·64] | 8·60e-03 |
| Group + Gender + Ejection Fraction | 5·76 [2·84 - 11·67] | 1·20e-06 | 3·30 [1·58 - 6·88] | 1·40e-03 |
| Group + Gender + Kilipp Score | 6·77 [3·52 - 13·02] | 9·50e-09 | 3·22 [1·55 - 6·70] | 1·80e-03 |
| Group + Gender + Serum Creatinine | 7·33 [3·78 - 14·19] | 3·50e-09 | 3·39 [1·62 - 7·06] | 1·10e-03 |
| Group + Gender + BNP | 5·88 [3·02 - 11·41] | 1·70e-07 | 2·55 [1·14 - 5·70] | 2·30e-02 |
| Group + Gender + NT-proBNP | 5·99 [3·10 - 11·57] | 1·00e-07 | 2·48 [1·14 - 5·37] | 2·20e-02 |
| Group + Diabetes + Ejection Fraction | 5·31 [2·61 - 10·83] | 4·40e-06 | 3·21 [1·43 - 7·16] | 4·50e-03 |
| Group + Diabetes + Kilipp Score | 6·25 [3·26 - 11·99] | 3·60e-08 | 3·04 [1·33 - 6·93] | 8·20e-03 |
| Group + Diabetes + Serum Creatinine | 6·85 [3·54 - 13·27] | 1·10e-08 | 3·27 [1·46 - 7·33] | 3·90e-03 |
| Group + Diabetes + BNP | 5·45 [2·82 - 10·56] | 4·90e-07 | 2·19 [1·04 - 5·18] | 4·80e-02 |
| Group + Diabetes + NT-proBNP | 5·60 [2·90 - 10·81] | 2·90e-07 | 2·17 [1·04 - 4·99] | 4·70e-02 |
| Group + Ejection Fraction + Kilipp Score | 5·42 [2·70 - 10·89] | 2·10e-06 | 3·44 [1·63 - 7·25] | 1·10e-03 |
| Group + Ejection Fraction + Serum Creatinine | 5·50 [2·68 - 11·31] | 3·40e-06 | 3·57 [1·70 - 7·48] | 7·60e-04 |
| Group + Ejection Fraction + BNP | 4·99 [2·43 - 10·24] | 1·20e-05 | 2·45 [1·06 - 5·65] | 3·60e-02 |
| Group + Ejection Fraction + NT-proBNP | 5·04 [2·45 - 10·37] | 1·10e-05 | 2·45 [1·13 - 5·30] | 2·30e-02 |
| Group + Kilipp Score + Serum Creatinine | 6·67 [3·46 - 12·85] | 1·40e-08 | 3·34 [1·58 - 7·09] | 1·70e-03 |
| Group + Kilipp Score + BNP | 5·37 [2·76 - 10·44] | 7·50e-07 | 2·47 [1·08 - 5·66] | 3·20e-02 |
| Group + Kilipp Score + NT-proBNP | 5·54 [2·86 - 10·72] | 3·90e-07 | 2·42 [1·11 - 5·28] | 2·60e-02 |
| Group + Serum Creatinine + BNP | 6·03 [3·09 - 11·77] | 1·40e-07 | 2·55 [1·12 - 5·78] | 2·50e-02 |

| | | | | |
|---|---|---|---|---|
| Group + Serum Creatinine + NT-proBNP | 6·16 [3·16 - 12·00] | 8·90e-08 | 2·50 [1·15 - 5·45] | 2·10e-02 |
| Group + BNP + NT-proBNP | 5·78 [2·95 - 11·32] | 3·00e-07 | 2·61 [1·20 - 5·66] | 1·50e-02 |

Subhazard ratios (SHR) and the *P* values associated to the effect of the group variable in competing risk model on the occurrence of HF (with death for all causes as adverse event) in both cohorts.
The variables in bold indicate the less favorable adjustment for the group variable.

**Table S6.** GO analysis of the 50 proteins selected in Table 2**, related to Tables 2 and 4**.

| ID GO | Description | Gene ratio | *P* value | Adjusted *P* Value | Uniprot ID* | Network # |
|---|---|---|---|---|---|---|
| 0007218 | Neuropeptide signaling pathway | 5/44 | 4·45E-06 | 0·006642 | | |
| 0043393 | Regulation of protein binding | 5/44 | 0·0001521 | 0·028386 | O60260, **PRKN** | 1 |
| | | | | | P56704, **WNT3A** | 2 |
| | | | | | P61769, **B2M** | 1 |
| | | | | | Q15848, **ADIPOQ** | 2 |
| | | | | | Q9Y644, RFNG | - |
| 0052547 | Regulation of peptidase activity | 7/44 | 8·22e-05 | 0·020365 | P56704, **WNT3A** | 2 |
| | | | | | P01042, **KNG1** | 1 |
| | | | | | P01034, **CST3** | 8 |
| | | | | | P05543, **SERPINA7** | 1 |
| | | | | | P01008, **SERPINC1** | 9 |
| | | | | | P29622, **SERPINA4** | 2 |
| | | | | | P19823, **ITIH2** | 2 |

* Selected molecules are in bold font (see Table 2)

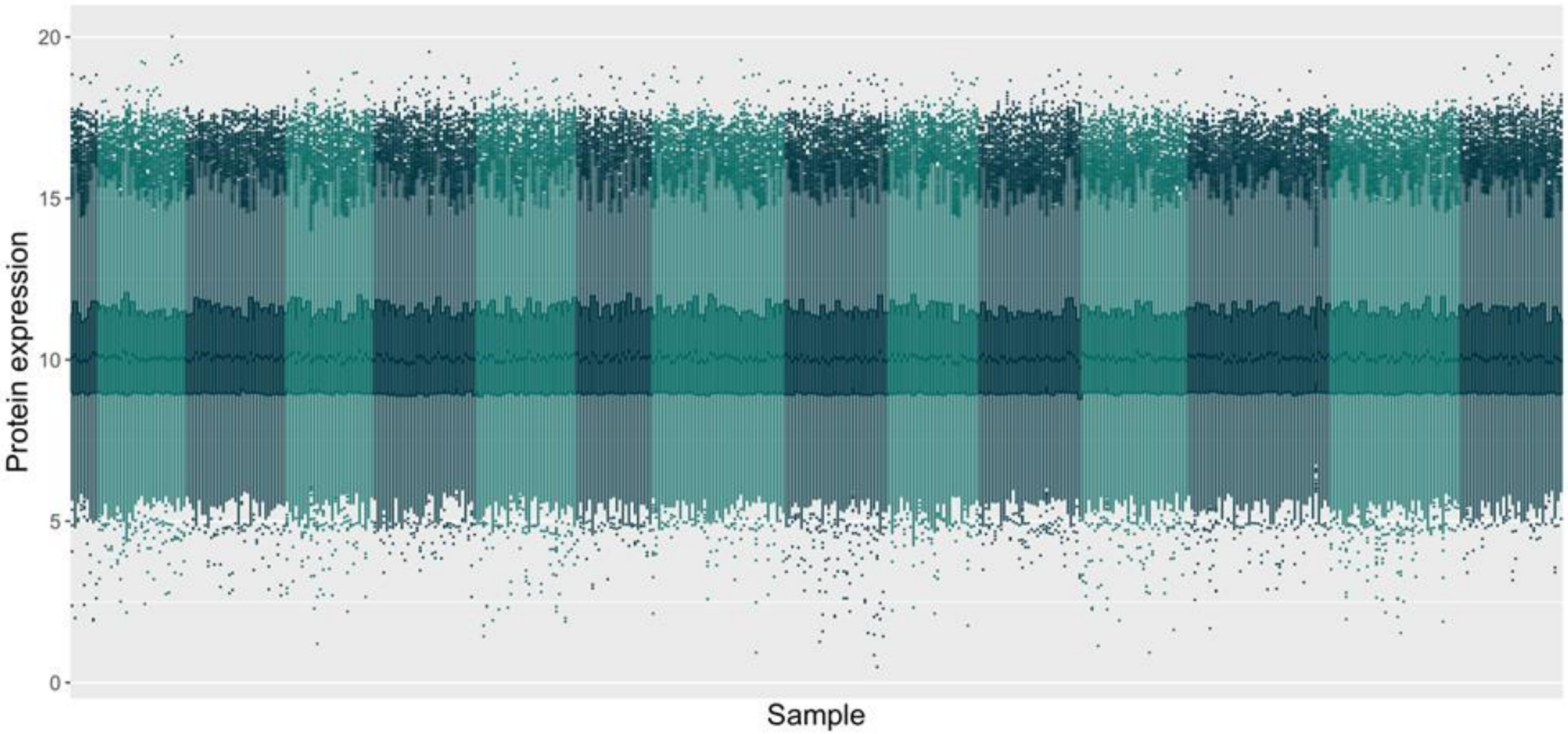


**Figure S1. Interplate variation in SomaScan assay, related to Table2.** Each boxplot (represented vertically) represents all the measured proteins for one sample. Each group of same colored boxplots corresponds to the same plate. In total, 15 plates were used.

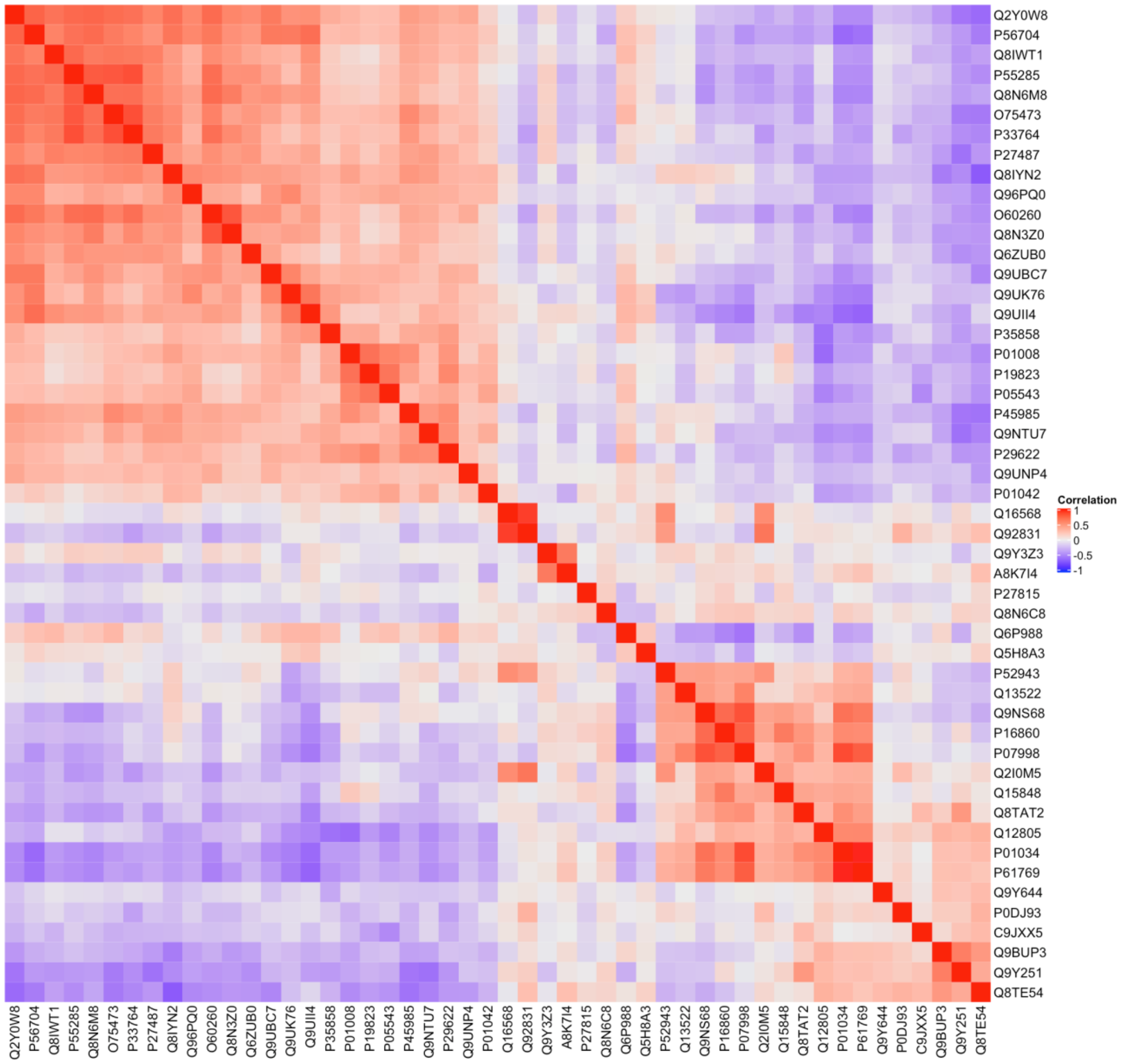


**Figure S2. Heatmap of the correlation matrix of the 50 selected proteins in REVE-1, related to Table2.** Each color cell represents the correlation between two proteins with highly correlated pairs of proteins in red and lowly correlated pairs of proteins in blue.

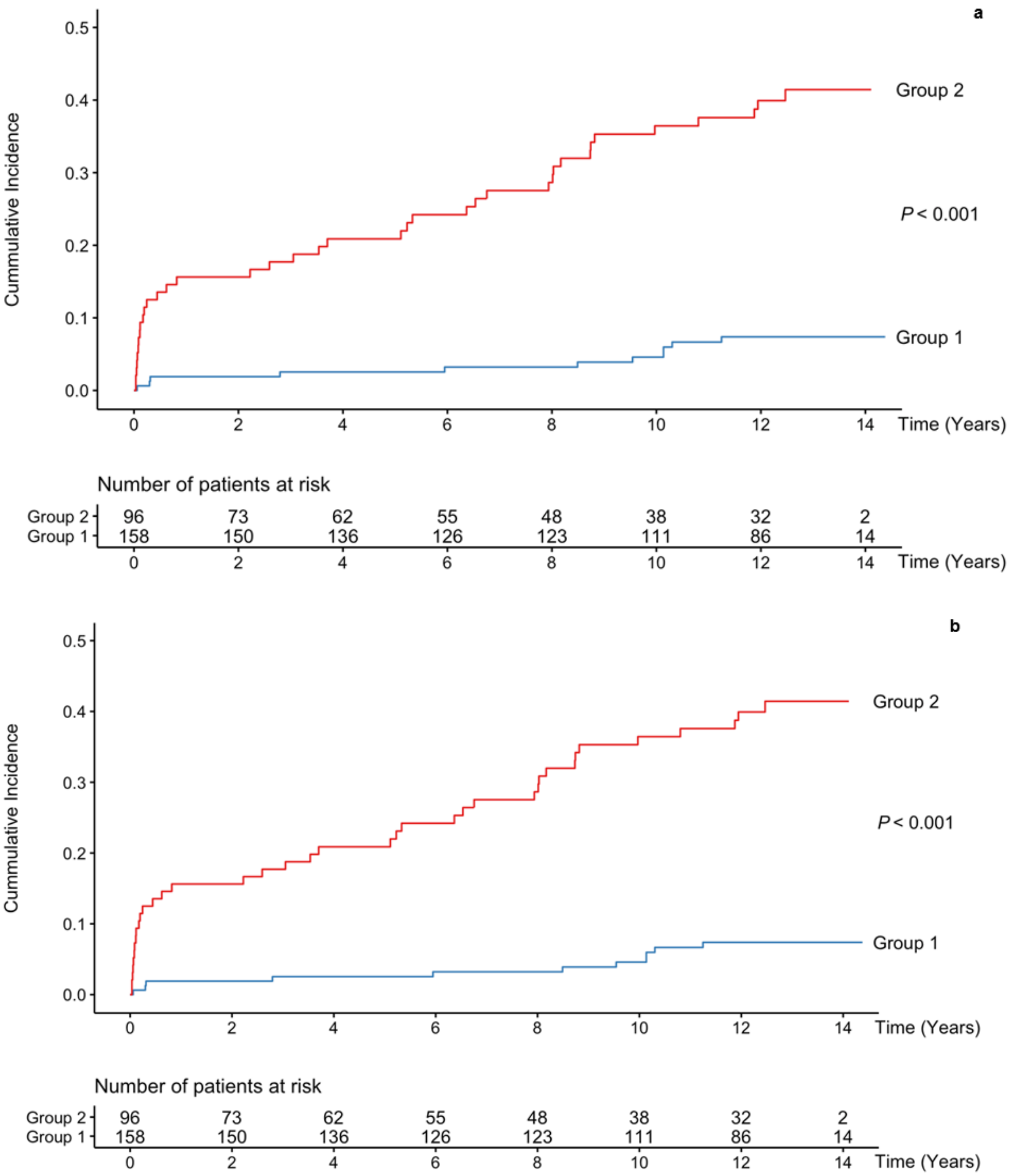


**Figure S3. Cumulative incidence curves of HF for patients from REVE-1 (a) and REVE-2 (b) cohorts with groups built without BNP, related to Figure 3**. The studied populations are divided in the two identified groups by clustering with the *k*-means performed on the 50 selected proteins minus the BNP. The group variable showed an SHR of 7.04 ([3.63 - 13.66], *P* <0.001) in REVE-1 and 3.82 ([1.83 - 8.00], P <0.001) in REVE-2, very similar to those obtained with all the 50 proteins (Figure 3).

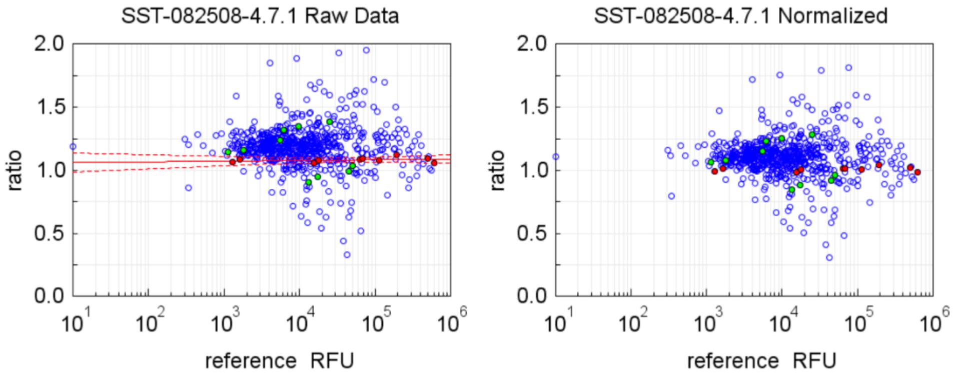


**Note Figure S4. Scatter plot for sample ratios versus their reference RFU for Hybridization Control Normalization.** The red filled circles are the RFU ratios for the hybridization controls fit with a regression line (red) displayed on the left plot prior to normalization. The inverse of the median ratio of the hybridization controls, 1/1.07, defines the scale factor = 0.935. The normalized data to which the 0.93 scale factor has been applied is displayed on the right plot. All ratios are decreased by application of the common scale factor and the controls now have a median ratio of one.

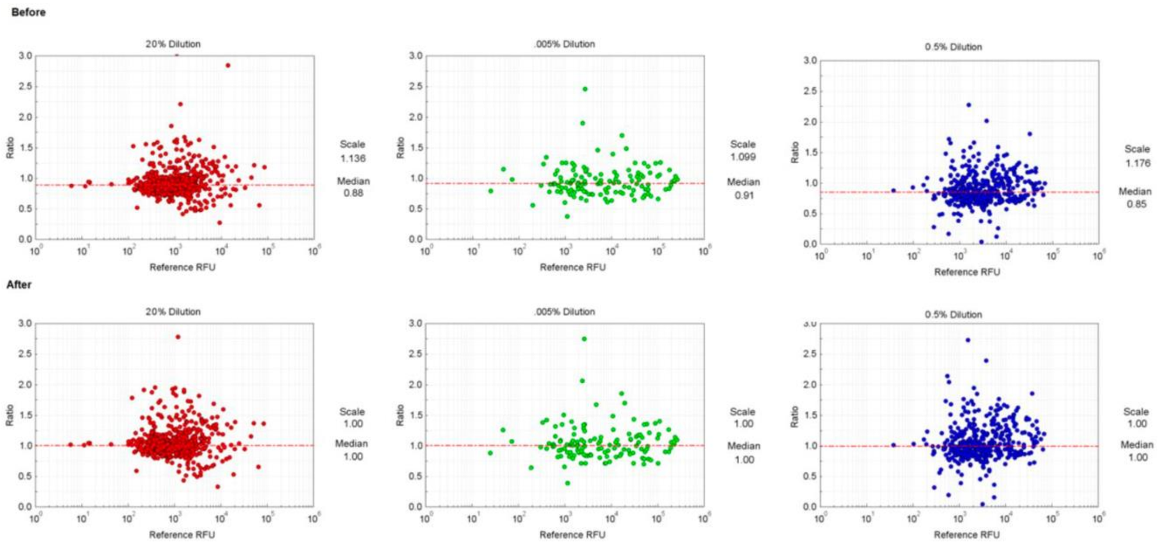


**Note Figure S5. Scatter plots for sample ratios versus their reference RFU for median signal normalization.** The filled circles are the RFU ratios for the SOMAmer binding reagents within a single dilution mix. The inverse of median ratios (1/0.88, 1/0.91 1/0.85) define the scale factors (1.136, 1.099, 1.176) applied to all the SOMAmer reagent measurements in the dilution mix, 20%, 0.005%, 0.5% respectively, for this sample. The ratios for the normalized data are displayed on the right. After normalization all ratios within the dilution mix are changed by application of the common scale factor and the SOMAmer reagents now have a median ratio of one, although the overall profile of the ratios defined by their relative distances has not changed.

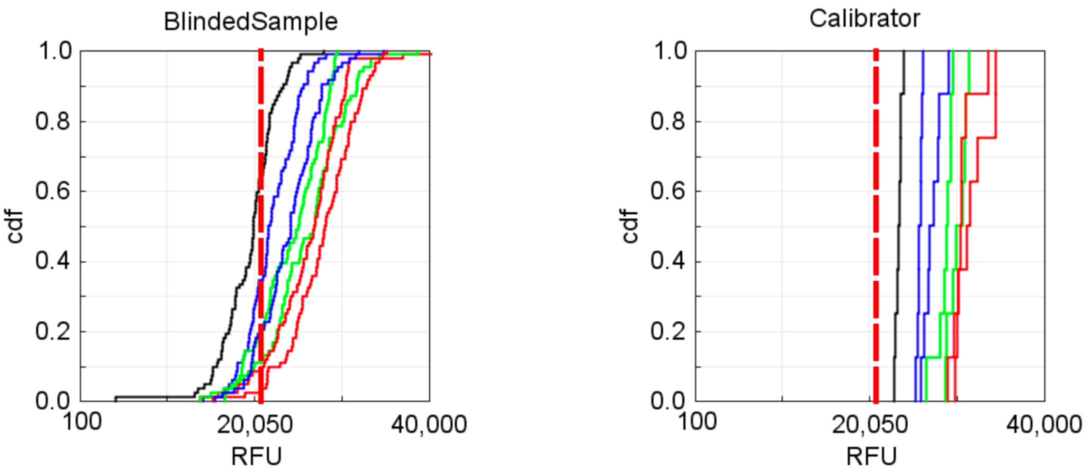


**Note Figure S6. Sample distributions illustrating systematic bias between assay plates.** Cumulative distribution functions (cdf) were generated for a single SOMAmer measurement across seven independent assay plates (approximately 600 samples randomly distributed across the plates) and are color coded by plate (left plot). The cdfs for the replicate calibrator sample measurements for each plate are displayed on the right for that SOMAmer reagent, color coded as on the left. The vertical red bar is the calibrator global reference obtained from a separate independent set of calibrator plates and is the target calibration RFU for this SOMAmer reagent's measurements. Note correlation of shifts between the clinical sample cdfs and the calibrator sample cdfs.

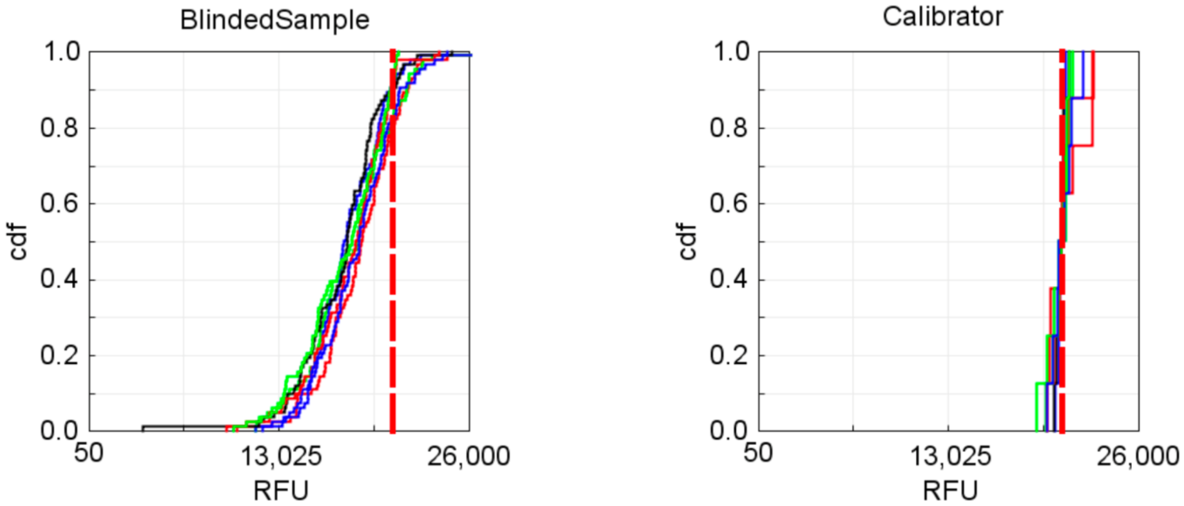


**Note Figure S6. Sample distributions illustrating removal of systematic bias between assay plates.** Cumulative distribution functions (cdf) for a set of clinical samples from Figure 3 after calibration (left plot). The cdfs for the replicate calibrator sample measurements for that SOMAmer reagent are displayed on the right. The median of each calibrator sample distribution equals the calibrator global reference standard by definition. Note the collapse of the clinical sample distributions to essentially a single distribution after calibration.
